# Integrative Analysis of Gene Expression Data by RNA Sequencing for Differential Diagnosis of Acute Leukemia: Potential Application of Machine Learning

**DOI:** 10.3389/fonc.2021.717616

**Published:** 2021-08-23

**Authors:** Jaewoong Lee, Sungmin Cho, Seong-Eui Hong, Dain Kang, Hayoung Choi, Jong-Mi Lee, Jae-Ho Yoon, Byung-Sik Cho, Seok Lee, Hee-Je Kim, Myungshin Kim, Yonggoo Kim

**Affiliations:** ^1^Department of Laboratory Medicine, College of Medicine, The Catholic University of Korea, Seoul, South Korea; ^2^Catholic Genetic Laboratory Center, Seoul St. Mary’s Hospital, College of Medicine, The Catholic University of Korea, Seoul, South Korea; ^3^Delvine Inc., Seoul, South Korea; ^4^Next Generation Sequencing (NGS) Division, Theragen Bio Co. Ltd., Seongnam-si, South Korea; ^5^Department of Hematology, Catholic Hematology Hospital and Leukemia Research Institute, Seoul St. Mary’s Hospital, College of Medicine, The Catholic University of Korea, Seoul, South Korea

**Keywords:** *BCR-ABL1*, mixed-phenotype acute leukemia, RNA sequencing, gene fusion, mutation, expression, machine learning, acute leukemia

## Abstract

*BCR-ABL1*–positive acute leukemia can be classified into three disease categories: B-lymphoblastic leukemia (B-ALL), acute myeloid leukemia (AML), and mixed-phenotype acute leukemia (MPAL). We conducted an integrative analysis of RNA sequencing (RNA-seq) data obtained from 12 *BCR-ABL1*–positive B-ALL, AML, and MPAL samples to evaluate its diagnostic utility. RNA-seq facilitated the identification of all p190 *BCR-ABL1* with accurate splicing sites and a new gene fusion involving *MAP2K2*. Most of the clinically significant mutations were also identified including single-nucleotide variations, insertions, and deletions. In addition, RNA-seq yielded differential gene expression profile according to the disease category. Therefore, we selected 368 genes differentially expressed between AML and B-ALL and developed two differential diagnosis models based on the gene expression data using 1) scoring algorithm and 2) machine learning. Both models showed an excellent diagnostic accuracy not only for our 12 *BCR-ABL1*–positive cases but also for 427 public gene expression datasets from acute leukemias regardless of specific genetic aberration. This is the first trial to develop models of differential diagnosis using RNA-seq, especially to evaluate the potential role of machine learning in identifying the disease category of acute leukemia. The integrative analysis of gene expression data by RNA-seq facilitates the accurate differential diagnosis of acute leukemia with successful detection of significant gene fusion and/or mutations, which warrants further investigation.

## Introduction

Next-generation sequencing (NGS) has been continuously expanded for use in clinical laboratories. It is now commonly used to detect gene mutations in DNA samples and identify recurrent fusions of RNA samples from cancer tissues using applicable cancer panels. Massive parallel sequencing methods using NGS panels are established clinical laboratory tests, which facilitate the detection of significant genetic changes. NGS panels usually include hundreds of genes, but they cannot identify genetic aberrations in unexpected genes. Therefore, recent studies have investigated the application of more extensive NGS platforms such as sequencing of whole genome, whole exome, and transcriptome for clinical cancer genomic profiling (1). Based on such extensive NGS platforms, novel disease categories were defined and recommended. The most representative example is *BCR-ABL1*-like acute lymphoblastic leukemia (ALL), which was first identified *via* hierarchical clustering of gene expression profile and a majority of them include gene fusions involving *CRLF2*, *JAK2*, and ABL gene categories (2). RNA sequencing (RNA-seq) was routinely used to classify *BCR-ABL1*-like ALL because it provided transcriptome data including gene expression profiling as well as gene fusions (3). Currently, RNA-seq is extensively used to analyze overall genomic data. Recent studies have improved the utility of RNA-seq in identifying gene mutations underlying various cancers, including hematologic malignancies (4, 5).

In this study, we performed RNA-seq of acute leukemia samples to evaluate its diagnostic utility. We intentionally selected *BCR-ABL1*–positive cases, which are recurrent gene fusions found in three categories of acute leukemia: B-lymphoblastic leukemia (B-ALL), acute myeloid leukemia (AML), and mixed-phenotype acute leukemia (MPAL). Although all such cases carry the disease-causing *BCR-ABL1* fusion, they differ in morphology and antigen expression. Each antigen represents specific hematopoietic lineages, which are usually analyzed by flow cytometry. First, we evaluated the analytical ability of RNA-seq to detect gene fusion and significant mutations. We then analyzed the gene expression data in order to select genes that are differentially expressed between disease categories and identify disease-specific pathways. In addition, we expanded the usage of gene expression data to identify the different disease categories based on the premise that mRNA expression reflects not only disease-specific pathways but also the hematopoietic lineage-associated antigen expression. Toward this end, we developed two models of differential diagnosis based on scoring algorithms and machine learning and verified using public datasets.

## Methods

### Patients and Samples

We reviewed medical records of patients who were diagnosed with acute leukemia and treated in Seoul St. Mary’s Hospital from February 2010 to March 2016. Standard diagnosis was established according to the WHO Classification of Tumours of Haematopoietic and Lymphoid Tissues based on bone marrow (BM) morphology, immunophenotyping, cytogenetic, and molecular genetic analysis (6). Among the consecutive cohorts, 349 patients were *BCR-ABL1–*positive including B-ALL (n = 224, 64.2%), AML (n = 10, 2.9%), and MPAL (n = 9, 2.6%). We selected 12 samples carrying p190 *BCR-ABL1* fusions including B-ALL (n = 5), AML (n = 3), and MPAL (n = 4) for further experimental investigation. Their clinical and laboratory characteristics are summarized in **Table 1**.

**Table 1:** Characteristics of patients at diagnosis.

Case	Sex/Age	WBC(10^9^/L)	BM blast(%)	Positive immunophenotype	Karyotype
ALL1	F/70	13,620	92	CD10, CD19, CD20, CD22, Cy-CD79a, CD34, HLA-DR	46,XX,t(9;22)(q34;q11.2)[1]/45,idem,-7,add(19)(p13.3)[8]/ 46,XX[11]
ALL2	F/50	3,570	99	CD10, CD19, CD22, Cy-CD79a, CD33, CD34, HLA-DR	46,XX,t(9;22)(q34;q11.2)[2]/ 46,idem,der(20)t(1;20)(q23;q13.1)[10]/46,XX[8]
ALL3	F/35	172,530	97	CD10, CD19, CD20, CD22, Cy-CD79a, CD33, CD34, HLA-DR	46,XX,t(3;22;9)(p25;q11.2;q34)[20]
ALL4	M/52	50,280	80	CD10, CD19, CD20, CD22, Cy-CD79a, CD34, HLA-DR	46,XY,der(9)del(9)(p13p22)t(9;22)(q34;q11.2),der(22)t(9;22)[12]/46,idem,del(11)(q11)[7]/46,XY[1]
ALL5	F/52	9,260	90	CD10, CD19, CD33, Cy-CD79a, CD34, HLA-DR	45,XX,der(3;7)(q10;q10),t(9;22)(q34;q11.2)[15]/46,XX[5]
AML1	F/57	89,170	75	CD33, CD11c, CD14, CD64, CD117, Cy-MPO, CD10, CD19, CD56, CD34, HLA-DR	46,XX,t(9;22)(q34;q11.2)[25]/46,XX[5]
AML2	M/29	55,530	73	CD33, CD11c, CD117, cy-MPO, CD19, CD34, HLA-DR	46,XY,t(9;22)(q34;q11.2)[20]
AML3	F/57	78,670	63	CD13, CD33, CD11c, CD14, CD64, Cy-MPO, CD10, CD19, CD22, CD34, HLA-DR	47,XX,+der(3;17)(q10;q10),t(9;22;14)(q34;q11.2;q32)[20]
MPAL1	M/33	23,290	99	CD10, CD19, CD79a, Cy-CD22, CD13, CD33, cy-MPO, CD7, CD34, HLA-DR	45,XY,-7,t(9;22)(q34;q11.2)
MPAL2	M/58	16,510	58	CD10, CD19, CD79a, Cy-CD22, CD13, CD33, CD11c, CD64, Cy-MPO, CD7, CD34, HLA-DR	46,XY,t(9;22)(q34;q11.2)
MPAL3	F/56	6,820	83	CD19, CD20, CD79a, Cy-CD22, CD13, CD33, Cy-MPO, CD34, HLA-DR	49,XX,+X,+4,+8,t(9;22)(q34;q11.2),i(17)(q10)[5]/49,sl,add(4)(p16),-10,add(12)(p11.2),+mar[10]/49,sdl1,del(13)(q14)[2]/48,sdl2,-del(13q)[2]/50,sl,+8[2]/50,sl,der(3)t(3;?13)(q27;q13),+8[2]/46,XX[4]
MPAL4	F/52	43,390	87	CD10, CD19, CD22, Cy-CD79a, CD33, cy- MPO, CD7, CD34, HLA-DR	45,XX,-7,t(9;22)(q34;q11.2)[18]/46,XX[2]

WBC, while blood cell count; BM, bone marrow; ALL, acute lymphoblastic leukemia; MPAL, mixed-phenotype acute leukemia; AML, acute myeloid leukemia; cy-, cytoplasmic.

### RNA-Seq and Identification of Fusion and Mutation

RNAs were extracted from BM samples using RNeasy^®^ Mini Kit (Qiagen, Hilden, Germany). RNA quality was assessed *via* analysis of rRNA band integrity on an Agilent RNA 6000 Nano Kit (Agilent Technologies, Santa Clara, CA, USA). Prior to cDNA library construction, 1 µg of total RNA and magnetic beads with Oligo (dT) were used to enrich poly(A) mRNA. The purified mRNAs were disrupted into short fragments, and the double-stranded cDNAs were immediately synthesized. The cDNAs were subjected to end-repair poly(A) addition and connected with sequencing adapters using the TruSeq RNA Sample Prep Kit (Illumina, San Diego, CA, USA). The fragments automatically purified by BluePippin 2% agarose gel cassette (Sage Science, Beverly, MA, USA) were selected as templates for PCR amplification. The final library sizes and qualities were evaluated electrophoretically using an Agilent High Sensitivity DNA Kit (Agilent Technologies, Santa Clara, CA, USA) and the fragment size ranged between 350 and 450 bp. Subsequently, the library was sequenced using an Illumina HiSeq2500 sequencer (Illumina, San Diego, CA, USA) to obtain 100-bp paired-end reads.

For read mapping and processing, low-quality reads were filtered according to the following criteria: reads containing more than 10% of skipped bases (marked as ‘N’s), reads containing more than 40% of bases whose quality scores were less than 20, and reads with average quality scores less than 20 each. The whole filtering process was performed using in-house scripts. Filtered reads were then mapped to the human reference genome [Ensembl release 72 (7)] using TopHat, which is supported by Bowtie2 (8). To identify the fusion genes, three different tools [FusionMap (9), nFuse (10), and Chimerascan (11)] were applied to RNA-seq datasets. Finally, the common fusion genes detected by the three tools were considered as actual fusion genes in each patient. To identify variants, GenomeAnalysisTK (v.2.3.9) and Mills-and-1000G-gold.standard-INDELs.hg19 (12) were used for realignment. BaseRecalibrator Tool in Genome Analysis ToolKit (GATK) was used for base quality score recalibration, and variants were called using SNPiR. All variants were then annotated using SnpEff (v.4.1).

### Massive Parallel Sequencing for Mutation Detection

DNAs were extracted from BM samples using QIAamp DNA Mini Kit (Qiagen, Hilden, Germany). DNA quality was assessed with Qubit dsDNA HS Assay Kit (Thermo Fisher Scientific, Waltham, MA, USA). SM acute leukemia panel customized for Seoul St. Mary’s Hospital was used to validate the detected mutations *via* RNA-seq. The SM panel consists of 67 genes with 1,239 DNA amplicons (13). The library preparation was performed *via* automated processes using IonChef™ system according to the manufacturer’s instructions (Thermo Fisher Scientific, Waltham, MA, USA). Sequencing was performed on an Ion S5 Sequencer (Thermo Fisher Scientific, Waltham, MA, USA). Read mapping, variant calling, and variant annotation were performed using the Ion Torrent Server software. Sequenced reads were mapped to the human reference genome (hg19, Genome Reference Consortium, February 2009) using TMAP v5.2.2. The pathogenic impact on gene function of missense mutations was estimated using in silico prediction of Sorting Intolerant From Tolerant (SIFT) and Polyphen-2. Score <0.05 is predicted to be deleterious in SIFT, and score close to 1.0 is predicted to be damaging in PolyPhen-2.

### Functional Annotation by Gene Set Enrichment Analysis (GSEA) and Gene Ontology (GO)

The gene expression level was measured with Cufflinks v2.1.1 (14) using the gene annotation database of Ensembl release 72. The non-coding region was removed. Multiread correction and frag-bias-correct were used to improve the accuracy of measurement. Differentially expressed genes (DEGs) were identified using Cuffdiff tool with a statistically significant *q*-value (<0.05). First, we performed functional annotation of DEGs according to the GO Consortium (http://www.geneontology.org/index.shtml) by R package, goseq (15). GO database classifies genes according to the three categories of biological process (BP), cellular component (CC), and molecular function (MF) and annotates the function of the selected genes. *P* values < 0.001 were considered statistically significant. This ontology result was rechecked using the database for annotation, visualization, and integrated discovery (DAVID) bioinformatics tool (16). In addition, we performed GSEA to analyze the critical transcriptome pathways (17). Toward this end, the estimated expression levels were used in GSEA to determine the enrichment scores according to the ranked-ordered gene list. With the predefined gene sets of GSEA, a total of 167 Kyoto Encyclopedia of Genes and Genomes (KEGG) pathways were considered, and the pathways containing at least 15 genes were evaluated. The significant scores were computed using 1,000 nonparametric permutation test, and *P* values < 0.05 were considered as statistically significant.

### Integration of Gene Network Analysis

We uploaded the DEG lists containing gene identifiers (probe set IDs) and corresponding *P*-values to Ingenuity Pathway Analysis (IPA; Ingenuity Systems, www.ingenuity.com). The association of the gene sets with the canonical pathways indicated possible effects on the well-defined biological pathway of the IPA platform based on the most up-to-date knowledge base. We extracted the most drastically affected functions (*P*-value ≤ 0.05; |z-score|≥ 2). The right-tailed Fisher’s exact test was used to estimate the probability that the association between a set of molecules and a function or pathway might be due to random chance. We also generated protein-protein interaction networks and a network view, which revealed the molecular relationship between the molecules (18).

### Development of Differential Diagnosis Models; Scoring Algorithm and Machine Learning

The differential diagnosis of acute leukemia is based on antigen expression on the surface and in the cytoplasm, which are usually analyzed by flow cytometry. Because the antigen expression was closely associated with gene expression, we postulated that the three categories of acute leukemia can be distinguished *via* analysis of gene expression data. Therefore, we selected DEGs, which showed more than 2-fold expression and a significant *q*-value (<0.05) in AML compared with B-ALL, and defined them as AML-specific genes. B-ALL–specific genes were defined similarly. We limited genes with at least 100 fragments per kilobase of transcript per million mapped reads (FPKM). Utilizing these tools, we finally selected 251 AML- and 117 B-ALL–specific genes (**Supplementary Table S1**). Each gene showed a different FPKM value. Thus, it is necessary to set a threshold value to define whether or not the gene was positively expressed. We carefully set the threshold and developed an analytical approach for application of the model to any gene expression data such as microarray as well as RNA-seq of acute leukemia cases. The details of the process and the formula are described in the Supplementary Materials. When the FPKM value of one gene was greater than the final threshold, it was included in the number of positively expressed genes. We obtained the AML and B-ALL scores based on the proportion of positively expressed AML- and B-ALL–specific genes, respectively. As expected, the AML score was the highest in AML followed by MPAL- and B-ALL–specific genes, and the B-ALL score was in the reverse order (**Supplementary Figure S1A**). We classified AML, B-ALL, and MPAL based on two scores according to the following criteria: 1) AML when AML score ≥ 30 and B-ALL score < 30, 2) B-ALL when AML score < 30 and B-ALL score ≥ 30, and 3) MPAL when both AML and B-ALL scores ≥ 30. Repeated simulations were performed to optimize the differential diagnosis scoring algorithm to obtain a diagnostic accuracy of 92% (11/12, **Supplementary Figure S1B**).

In addition, we conducted machine learning for differential diagnosis using gene expression data selected for the scoring algorithm. The soft margin support vector machine (SVM) (19) was used because the gene expression data were not linearly separable. Kernel function of hyperbolic tangent was applied for SVM. The Z-transformation of each disease category was carried out for standardization. We selected the one-vs.-one strategy, which splits multiple classes into single binary elements for each pair of classes (e.g., B-ALL-vs.-AML). Although the sample size was small to generate enough datasets for machine learning, we performed one-vs.-all SVM cross-validation to reduce this limitation. This method repeated the analyses using data from 1 case for validation and the other 11 for learning, which is described in the Supplementary Materials. The cross-validation of our SVM model of machine learning based on our 12 cases revealed 100% diagnostic accuracy, sensitivity, and specificity.

### Validation of Differential Diagnosis Models Using Public Datasets

To validate the models for differential diagnosis of acute leukemia, we collected 427 public gene expression datasets from the International Cancer Genome Consortium (ICGC) Data Portal (dcc.icgc.org; 206 samples of ALL-US project EXP-A data and 197 samples of LAML-US project EXP-A data) and the National Center for Biotechnology Information (NCBI) website (www.ncbi.nlm.nih.gov; 24 samples of Series GSE113601 dataset). Reference gene (*ABL1*) was used as the internal reaction control to normalize the gene expression. AML and B-ALL scores were calculated as described previously. We also validated the performance of a machine learning method using normalized gene expression levels of the same public dataset.

### Statistical Analysis

Statistical differences between groups were determined using one-way analysis of variance (ANOVA) followed by Bonferroni’s *post hoc* test for multiple comparisons. Pearson’s correlation analysis was performed for the quantitative results of variant allele frequencies (VAFs) from RNA-seq with those from massive parallel sequencing. All analyses were performed using IBM^®^ SPSS^®^, version 24.0 (IBM Corp., Armonk, NY, USA). Differences were significant at *P* < 0.05.

## Results

### Detection of the Gene Fusions and Mutations by RNA-Seq

RNA-seq revealed *BCR-ABL1* gene fusions in all enrolled cases. The number of fusion transcripts varied in each case: 1 to 4 for *BCR-ABL1* and 0 to 3 for *ABL1-BCR*. All cases carried a *BCR-ABL1* fusion transcript with a splicing site of chr22:23524426 and chr9:133729451. The secondary splicing site located on the three base pairs differed from the first *ABL1* splicing site as chr9:133729454, which was observed in five cases. *ABL1-BCR* fusion transcripts were detected in 10 cases with a shared splicing site in chr9:133589842 (exon 1a) and chr22:23595986. The second common splicing site was located on chr9:133710912 (exon 1b) with the same *BCR* splicing site. No differences in splicing sites were detected in cases according to the disease category (**Supplementary Figure S2**).

In addition, we found a new fusion in a B-ALL patient with der(19)t(7;19)(p14;p13.3); mitogen-activated protein kinase kinase 2 (*MAP2K2*)-AC010132.5. The *MAP2K2* gene, located on 19p13.3, is a dual-specificity protein kinase belonging to the MAP kinase kinase family. This kinase is known to play a critical role in mitogen growth factor signal transduction. AC010132.5 is located on7p14. This fusion was confirmed by Reverse Transcription Polymerase Chain Reaction (RT-PCR) and Sanger sequencing (**Supplementary Figure S3**).

DNA-based SM NGS panel detected 11 single-nucleotide variations, two insertions, and one in-frame deletion. RNA-seq detected these variants except two on the *NOTCH3* gene because of the low gene expression value as FPKM < 1.0 (**Table 2**). VAFs from RNA-seq showed good correlation with those from massive parallel sequencing except mutations with low FPKM < 5.0 (r = 0.787, *P* = 0.004). We also analyzed the point mutations within the *ABL1* kinase domain associated with drug resistance and found no significant mutation.

**Table 2:** Mutations analyzed by massive parallel sequencing of DNA and RNA sequencing.

Case	Gene	Transcript	Base change	AA Change	Type of mutation	VAF^*^	RNA-seq	VAF^**^	FPKM^**^	SIFT^†^	Polyphen-2^†^
ALL1	*SETD2*	NM_014159.6	c.1409_1410insGCCC	R471Pfs*21	frameshift	29.45	Detected	33.33	40.51	–	–
ALL3	*PAX5*	NM_016734.2	c.55_56insTG	G19Vfs*3	frameshift	38.34	Detected	29.81	238.84	–	–
ALL4	*NOTCH3*	NM_000435.2	c.3736G>A	V1246I	missense	50.33	Not detected	–	0.4	0.52	0
ALL4	*TET2*	NM_001127208.2	c.3116C>T	S1039L	missense	53.55	Detected	34.48	10.6	0.29	0.968
ALL4	*PAX5*	NM_016734.2	c.70G>C	G24R	missense	6.37	Detected	13.64	78.66	0	1.000
AML1	*NOTCH3*	NM_000435.2	c.709G>A	V237M	missense	52.36	Detected	–	1.04	0	0.908
AML2	*NOTCH3*	NM_000435.2	c.33_35delCCG	R12del	in-frame deletion	4.62	Not detected	–	0.79	–	–
AML2	*RUNX1*	NM_001754.4	c.610C>T	R204*	nonsense	43.35	Detected	52.94	105.24	–	–
AML2	*RUNX1*	NM_001754.4	c.601C>T	R201*	nonsense	45.35	Detected	44.20	105.24	–	–
AML3	*NF1*	NM_001042492.2	c.4379A>G	H1460R	missense	65.3	Detected	68.57	27.73	0.27	0.048
MPAL2	*TET2*	NM_001127208.2	c.2604T>G	F868L	missense	53.33	Detected	63.64	14.54	0.22	0.307
MPAL2	*NF1*	NM_001042492.2	c.1198C>G	Q400E	missense	45.9	Detected	61.54	6.1	1	0.008
MPAL4	*SH2B3*	NM_005475.2	c.1139T>C	L380P	missense	47.95	Detected	42.86	39.96	0.2	0.966
MPAL4	*NOTCH3*	NM_000435.2	c.709G>A	V237M	missense	48.29	Detected	66.67	1.09	0	0.908

RNA-seq detected most variants detected in DNA sequencing except two with low gene expression value as FPKM < 1.0.

AA, amino acid; VAF, percentage of variant allele frequency; RNA-seq, RNA sequencing; FPKM, Fragment Per Kilobase of transcript per Million mapped reads; SIFT, Sorting Intolerant From Tolerant.

^*^VAF obtained from massive parallel sequencing of DNA.

^**^VAF and FPKM obtained from RNA-seq.

^†^The SIFT and Polyphen2 in-silico scores are unavailable for frameshift and nonsense mutations and in-frame deletion.

### Different Gene Expression Profiles Among AML, B-ALL, and MPAL

**Figure 1:**
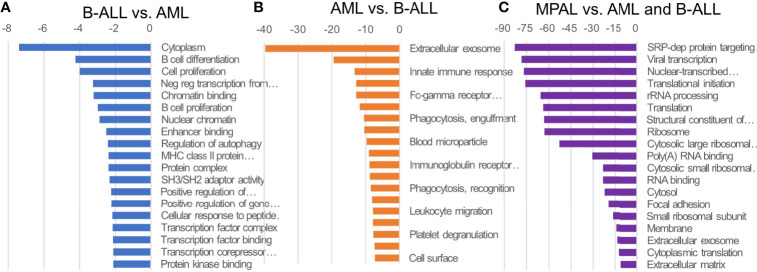
GO of ALL, AML, and MPAL. General functional classification of highly expressed gene in B-lymphoblastic leukemia (B-ALL) compared to acute myeloid leukemia (AML) **(A)**, AML compared to B-ALL **(B)**, and in mixed-phenotype acute leukemia (MPAL) compared to B-ALL and AML **(C)**. Gene Ontology (GO) analysis within target genes of significantly altered transcripts was performed using the database for annotation, visualization and integrated discovery (DAVID) bioinformatics tool. Enriched GO biological processes were identified and listed according to their enrichment P value (P < 0.05) and false discovery rate (FDR < 0.25). Both P and FDR values were obtained using DAVID 2.1 statistical function classification tool. scale: -log_10_ of p-value.

We identified significantly different GO profiles among the three disease categories including mitogen-activated protein kinase (MAPK) cascade, angiogenesis, cell activation, cytokine production, regulation of protein phosphorylation, immune system process, leukocyte differentiation, receptor activity, receptor binding, cell communication and adhesion, and cell death and proliferation (**Supplementary Table S2**). When comparing AML with B-ALL, the genes associated with extracellular exosome, extracellular space, innate immune response, defense response to bacterium, Fc-gamma receptor signaling pathway involved in phagocytosis, plasma membrane, phagocytosis, and engulfment serine-type endopeptidase activity were enriched in AML. The genes associated with cytoplasm, B cell differentiation, and cell proliferation were enriched in B-ALL. Compared with the other leukemias, MPAL revealed higher gene expression of RNA- and translation-related ontologies (**Figure 1**).

In terms of GSEA, the KEGG pathway analyses of 167 gene sets showing significantly different expression between AML and B-ALL identified 118 upregulated gene sets in AML and 49 upregulated in B-ALL. Among the upregulated gene sets in AML, 29 gene sets showed false discovery rate (FDR) < 0.25 and a nominal *P* value < 0.01 (**Supplementary Table S3**). Among the upregulated gene sets in B-ALL, two gene sets showed significant FDR and nominal *P* value. The IPA analysis of these gene sets indicated the major altered canonical pathways in MPAL including IL-6 signaling, PPAR signaling, 14-3-3–mediated signaling, osteoarthritis pathway, D-myo-inositol-5-phosphate metabolism, antioxidant action of vitamin C, regulation of the epithelial mesenchymal transition by growth factors pathway, super pathway of inositol phosphate compounds, cardiac hypertrophy signaling (enhanced), and PI3K signaling in B lymphocytes (**Supplementary Figure S4**).

### Predictive Performance of Developed Differential Diagnosis Models

**Figure 2:**
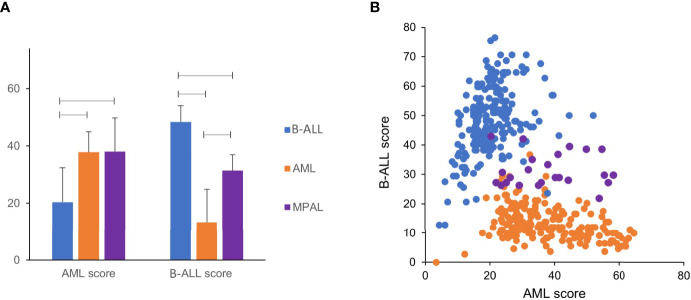
AML score and B-ALL score of public data. **(A)** Except AML score between AML and MPAL, AML score and B-ALL score between each leukemia were significantly different (p < 0.01). **(B)** Public data of AML and B-ALL show clustering in scatter plot of AML score and B-ALL score. MPAL samples cluster between AML and B-ALL samples.

We analyzed the public gene expression datasets and compared the AML and B-ALL scores of three disease categories. The AML score was significantly high in AML and MPAL compared with B-ALL (*P* < 0.001 and < 0.001, respectively). The B-ALL score was significantly high in B-ALL followed by MPAL and AML (*P* < 0.001 for each comparison) (**Figure 2A**). In the scattergram of B-ALL score and AML score, the MPAL samples cluster between AML and B-ALL samples (**Figure 2B**). The diagnostic accuracy of scoring algorithm and machine learning was 97.2% (415/427) and 99.1% (423/427), respectively. We analyzed additional predictive performance of two differential diagnosis models according to disease category. The diagnostic sensitivities of the scoring algorithm were 99.0%, 95.4%, and 95.8% in B-ALL, AML, and MPAL, respectively. Those of the machine learning were 99.5%, 99.5%, and 91.7% in the same order, respectively. The specificities of the scoring algorithm were 95.5%, 98.7%, and 97.3% in B-ALL, AML, and MPAL, respectively. And those of the machine learning were 100%, 98.7%, and 99.8% in the same order, respectively (**Supplementary Table S4**).

## Discussion

In the current study, we performed RNA-seq of 12 acute leukemia cases carrying p190 *BCR-ABL1*. The analysis of three disease categories using various angles facilitated the comprehensive evaluation of RNA-seq performance in gene fusion, mutation, and gene expression and the application of expression data to differential diagnosis. RNA-seq not only enabled the reliable detection of all *BCR-ABL1* but also provided accurate splicing sites. There was a common splicing site of *BCR* and *ABL1* genes as chr22:23524426 and chr9:133729451. Some patients carried more than one *BCR*-*ABL1* fusions. *ABL1*-*BCR* fusion was generated in some patients. Notably, a new fusion was detected in this study. Gene fusions in acute leukemia are implicated in the onset of malignancies, and recurrent driver gene fusions are used in the classification. Novel gene fusions have been continuously discovered in hematologic malignancies and represent potential diagnostic or therapeutic targets (20, 21). RNA-seq is a useful tool for genome-wide surveillance of gene fusions with nucleotide-level resolution of fusion junctions (22). Studies have demonstrated the potential to identify gene fusions *via* improved bioinformatics workflows (23–25), and therefore, RNA-seq can be used not only to identify driver gene fusions but also to detect novel and rare gene fusions in clinical laboratories.

Gene mutations are usually analyzed *via* DNA sequencing. However, we postulated that RNA-seq facilitates the screening of clinically significant mutations based on sequencing data. The current and previous studies have shown that somatic mutations can be identified in cancer based on RNA-seq data of cancer-related genes (4, 26). A specific software, i.e., RNAmut, which enabled the detection of all clinically important mutations in AML, was developed (5). And tandem duplications in *FLT3* and *KMT2A* were also effectively detected by RNA-seq using those novel algorithms (5, 27). Integrated genomic profiling has increased the ability to identify clinically relevant genomic alterations of therapeutic significance in hematologic malignancies (28). In the current study, we successfully detected clinically significant somatic mutations using RNA-seq data except the mutations in the genes with very low transcript. Also, their VAFs from RNA-seq showed good correlation with those from massive parallel sequencing. Additionally, we identified mutations within the *ABL1* kinase domain *via* RNA-seq, which was associated with drug resistance to tyrosine kinase inhibitors. However, the study showed an intrinsic limitation in that poorly expressed gene mutations could not be analyzed by RNA-seq.

Gene expression data showed significantly different GO profiles among the three disease categories. In B-ALL, B cell differentiation and transcription factor–associated GOs were enriched. The result was in agreement with previous studies that demonstrated that malignant conversion of B-lymphocyte progenitors involves multiple targeting of a central transcription factor network (29). In AML, GOs associated with functions of granulocytes were enriched such as phagocytosis, lysosome, and innate immune response. Also, results from this and previous studies showed that extracellular exosome was enriched in AML, which express the properties relevant to AML pathogenesis that affected prognosis, response to therapy, and leukemic niche formation (30). The MAPK cascade is one of the differential GO profiles that plays an essential role in connecting cell-surface receptors with altered transcriptional programs and aberrant MAPK activation in the pathogenesis of various myeloid malignancies (31). In addition, the stress-activated MAPK pathways influence the response of cancer cells to chemotherapies and targeted therapies (32). Therefore, the role of different ontology profiles in each disease category requires further elucidation.

>We investigated MPAL-specific pathways *via* substantial analysis of gene expression profiles and found out that MPAL constituted a heterogeneous group of diseases rather than induced by a specific pathway. MPAL is acute leukemia of ambiguous lineage that is defined by antigen expression. MPAL contains blasts that express antigens of more than one lineage to such a degree that is not possible to attribute the leukemia to any one lineage definitively. Other evidence suggests that the cell of origin in B-ALL associated with *BCR-ABL1* was more immature than that of other B-ALL cases (33). A hematopoietic progenitor cell with multilineage potential is considered as a normal counterpart of the leukemic cells in AML and MPAL with *BCR-ABL1* (33). Therefore, we carefully postulated that acute leukemia with *BCR-ABL1* may represent a series of diseases carrying different proportions of specific lineage.

This is the first trial to develop models of differential diagnosis using RNA-seq. Based on AML and ALL-specific gene expression data, we developed two models, namely, scoring algorithm and machine learning. Although there was a practical limitation of small sample size for machine learning, both models were effective not only in our 12 *BCR-ABL1–*positive cases but also in public datasets from acute leukemias regardless of specific genetic aberration. In addition, gene expression data contain profound information including molecular pathways associated with disease pathogenesis and potential therapeutic targets. Recently, the usage of gene expression data has been expanded by integrating traditional medical data and advanced rapidly using machine learning algorithms in real clinical medicine (13). The machine learning model discussed in this study might be used to integrate gene expression data with gene fusions and mutations.

Taken together, this study demonstrated that RNA-seq not only enabled the detection of gene fusion and clinically significant mutations but also assigned the lineage of acute leukemia according to gene expression. The potential application of gene expression data by RNA-seq data that facilitate the accurate differential diagnosis of acute leukemia requires further investigation.

## Data Availability Statement

RNA-seq data generated in this study is available at NCBI BioProject database: https://www.ncbi.nlm.nih.gov/bioproject/PRJNA733693. Gene expression data of ACUTE LYMPHOBLASTIC LEUKEMIA - TARGET, US is available at ICGC Data Portal: https://dcc.icgc.org/projects/ALL-US. Gene expression data of ACUTE MYELOID LEUKEMIA - TCGA, US is available at ICGC Dapa Portal: https://dcc.icgc.org/projects/LAML-US. Gene expression data of RNA sequencing analysis of adult mixed phenotype acute leukemia (MPAL) is available at NCBI GEO: https://www.ncbi.nlm.nih.gov/geo/query/acc.cgi?acc=GSE113601.

## Ethics Statement

The study protocol was approved by the Institutional Review Board of The Catholic University of Korea (No.KC16SISI1026) and performed in accordance with the Declaration of Helsinki.

## Author Contributions

MK and YK were responsible for the study concept and project administration. J-HY, B-SC, SL and H-JK acquired resources. JL, SC, S-EH, J-ML, DK and HC analyzed and interpreted data. JL drafted the manuscript. All authors contributed to the article and approved the submitted version.

## Funding

This research was funded by Ministry of Food and Drug Safety (grant number 18172MFDS182) and the National Research Foundation of Korea (NRF) funded by the Korea government (MSIT) (grant number 2020R1F1A1068437).

## Conflict of Interest

Author SC is the CEO of Delvine Inc. Author SH is employed by Theragen Bio Co. Ltd.

The remaining authors declare that the research was conducted in the absence of any commercial or financial relationships that could be construed as a potential conflict of interest.

## Publisher’s Note

All claims expressed in this article are solely those of the authors and do not necessarily represent those of their affiliated organizations, or those of the publisher, the editors and the reviewers. Any product that may be evaluated in this article, or claim that may be made by its manufacturer, is not guaranteed or endorsed by the publisher.
