# Supplementary material for: Integrative Analysis of Gene Expression Data by RNA Sequencing for Differential Diagnosis of Acute Leukemia: Potential Application of Machine Learning
Source: Front Oncol. 2021 Aug 23;11:717616. doi: 10.3389/fonc.2021.717616 (PMC8419339; doi:10.3389/fonc.2021.717616)
Supplement: Supplementary file 1 [file DataSheet_1.docx]

***Supplementary Material***

**Index**

1. Supplementary Methods
2. QC matrices
3. Supplementary Figures
4. Supplementary Tables
5. **Supplementary Methods**

**The differential diagnosis algorithm**

We defined the robust threshold of FPKM for each gene, which potentially minimized false determination. In three different scenarios (i, ii, and iii), FPKM showed more than 2-fold difference.

(i) (ii) (iii)


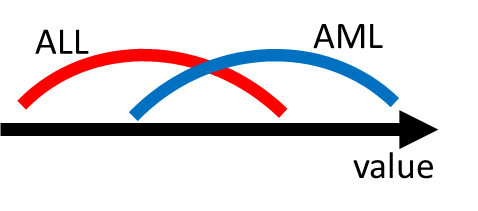

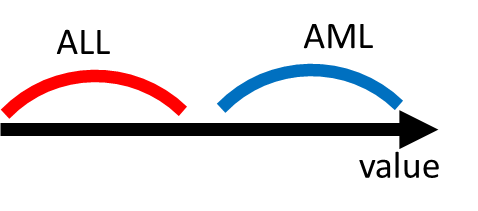

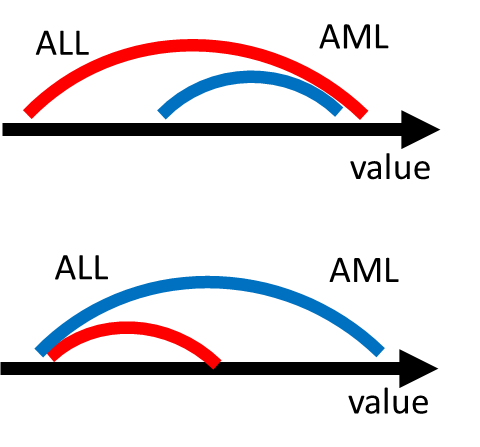


Because it was difficult to develop the individual threshold for each scenario, a formula was needed to interpolate the difference using a coefficient. The coefficient was intended to interpolate standard deviations of FPKM value in each disease category.

1. Primary coefficient(*α_i_*) of AML-specific genes

$$\alpha_{i} :\frac{\mathrm{std}(\boldsymbol{V}_{M,i})}{std\left( \boldsymbol{V}_{M,i} \right)+std\left( \boldsymbol{V}_{L,i} \right)}$$

$\boldsymbol{V}_{M,i} :FPKM value of i gene in AML$

$\boldsymbol{V}_{L,i} :FPKM value of i gene in ALL$

2. Primary coefficient(*α_i_*) of B-ALL-specific genes

$$\alpha_{i} :\frac{\mathrm{std}(\boldsymbol{V}_{L,i})}{std\left( \boldsymbol{V}_{M,i} \right)+std\left( \boldsymbol{V}_{L,i} \right)}$$

$\boldsymbol{V}_{M,i} :FPKM value of i gene in AML$

$\boldsymbol{V}_{L,i} :FPKM value of i gene in B-ALL$

Then, we developed tentative thresholds of FPKM for each gene as medians of AML samples and B-ALL samples.

- Tentative threshold = $\frac{median FPKM in AML samples + median FPKM in B-ALL samples}{2}$

Optimizing coefficient was calculated to resolve overfitting of threshold in AML and B-ALL.

$$Optimizing coefficient =$$

$$\frac{1}{N_{m+l}}\left( \sum_{i∊m} \frac{\alpha_{i}\min( \boldsymbol{V}_{M,i})+(1-\alpha_{i})max( \boldsymbol{V}_{L,i})}{({\mathrm{median}\left( \boldsymbol{V}_{M,i} \right)+\mathrm{median}\left( \boldsymbol{V}_{L,i} \right))}/2}+\sum_{i∊l} \frac{(1-\alpha_{i})max( \boldsymbol{V}_{M,i})+\alpha_{i}\min( \boldsymbol{V}_{L,i})}{({\mathrm{median}\left( \boldsymbol{V}_{M,i} \right)+\mathrm{median}\left( \boldsymbol{V}_{L,i} \right))}/2} \right)$$

$\boldsymbol{V}_{M,i} :FPKM value of i gene in AML$

$\boldsymbol{V}_{L,i} :FPKM value of i gene in B-\mathrm{ALL}$

$m :positively expressed genes in AML$

$l :positively expressed genes in B-ALL$

$N :the number of selected genes$

The optimizing coefficient calculated with our data was 0.8, which distinguished AML from B-ALL optimally, and vice versa. The final threshold of FPKM was set as follows.

Final threshold = tentative threshold $\times$ 0.8 (optimizing coefficient)

When the FPKM value of one gene was greater than the final threshold, it was included in a number of positively expressed genes. The AML and B-ALL scores were calculated as the number of positively expressed genes divided by the total number of available AML-specific or B-ALL-specific genes (percentages), respectively. Thus, we derived both AML and B-ALL scores in each case. We classified the disease category according to the following criteria; 1) AML when AML score ≥ 30 and B-ALL score < 30; 2) B-ALL when AML score < 30 and ALL score ≥ 30; and 3) MPAL when both AML and B-ALL scores ≥ 30. The scores and simulation results are presented in the following Table. All cases except an MPAL case were concordantly simulated as their original diagnosis. MPAL2 revealed a low B-ALL score, which thus simulated AML. The case showed the characteristics of acute bilineal leukemia with two distinct populations of myeloid and lymphoid lineages instead of biphenotypic leukemia involving other three MPAL cases.

|  | ALL1 | ALL2 | ALL3 | ALL4 | ALL5 | AML1 | AML2 | AML3 | MPAL1 | MPAL2 | MPAL3 | MPAL4 |
| --- | --- | --- | --- | --- | --- | --- | --- | --- | --- | --- | --- | --- |
| AML score | 10 | 14 | 12 | 13 | 7 | 98 | 89 | 88 | 34 | 74 | 43 | 74 |
| ALL score | 85 | 84 | 86 | 95 | 84 | 4 | 4 | 9 | 58 | 8 | 62 | 43 |
| Simulation | ALL | ALL | ALL | ALL | ALL | AML | AML | AML | MPAL | AML | MPAL | MPAL |

When we simulated 427 cases from public data using the diagnostic algorithm described above, we found that 97.2% showed concordance with the original diagnosis.

| Original diagnosis | | AML (n=197) | B-ALL (n=206) | MPAL (n=24) |
| --- | --- | --- | --- | --- |
| Simulation | AML | 188 | 2 | 1 |
|  | ALL | 7 | 204 | 0 |
|  | MPAL | 2 | 0 | 23 |

**Support Vector Machine**

A support vector machine (SVM) is a supervised machine learning model that uses classification algorithms for binary classification. The goal is to determine the class of a new data point. In SVM algorithm, we plot each data as a point in n-dimensional space, where n is the number of features (the number of selected genes in our case) with the value of each feature representing the value of a particular coordinate. The classification is based on the hyperplane clearly differentiating the two classes. Basically, the hyperplane is selected to maximize the distance from the hyperplane to the nearest data point in each class.

If the training data are linearly separable, two hyperplanes can be identified that are parallel and carry the largest distance for categorization into two classes. The region bound by these two hyperplanes is called the “margin”. When the distance of the margin is $\boldsymbol{2}/\left\| \mathbf{w} \right\|$**,** the goal of the classifier is to maximize the distance of the margin. In order to maximize margin, $\left\| \mathbf{w} \right\|$should be minimized, and the following equation is used to resolve constrained optimization.

$$\min\frac{1}{2}\left\| \mathbf{w} \right\|^{2}, s.t. y_{i}\left( w\cdot x_{i}+b \right)\geq1, i=1,\ldots..n$$

$$\mathbf{w, b}:coefficient of hyperplane , x_{i} :observed data point$$

The condition suggests that the predicted data and the existing label are classified under the same class. If a linear hyperplane with maximized margin is used, a soft margin is applied to the data that is not linearly separable. The value proportional to the distance between the hyperplanes of each class is added to the objective function to obtain the hyperplane with the maximum margin. Therefore, SVM with a soft margin can be used to identify a hyperplane that minimizes the distance and maximizes margin.

Additionally, for the use of nonlinear type of SVM, the feature space of the data was transformed to obtain maximum-margin hyperplane. In this study, the soft margin and kernel method were applied together. The objective function was as follows:

$$\min\frac{1}{2}\left\| \mathbf{w} \right\|^{2}+C\sum_{i=1}^{m} \zeta_{i}$$

$$s.t. y_{i}\left( w\cdot\phi\left( x_{i} \right)+b \right)\geq1- \zeta_{i}, i=1,\ldots..n, \zeta_{i}\geq0$$

$$k\left( x_{i},x_{j} \right)= \phi\left( x_{i} \right)\cdot\phi\left( x_{j} \right) :kernel function$$

$$k\left( x_{i},x_{j} \right)=\tanh\left( ax_{i}\cdot x_{j}+b \right) :hyperbolic tangent kernel$$

$$\phi\left( x_{j} \right) :transformed data point$$

$$\zeta_{i} :slick variable, Allow some instances to fall off the margin, but penalize them$$

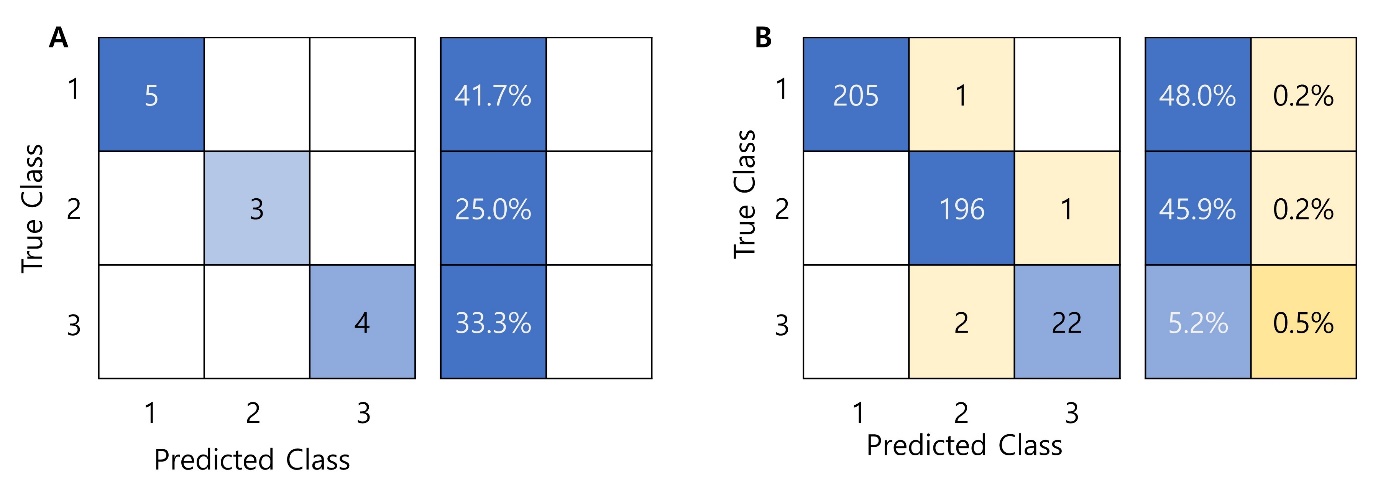
Confusion matrix based on cross-validation of our 12 cases (A) and public gene expression data (B)

**QC matrices in validated clinical samples**

After deleting the low-quality and adaptor sequences, we obtained approximately 50 to 53 million raw reads per sample, from which approximately 45 to 48 million clean reads were mapped to human reference genome (hg19), of which, approximately 40 to 45 million reads were mapped to genes. The number of mapped genes exceeded 16,000 per sample, and most of them were known genes, including nearly 75% showing over 60% coverage and about 45% over 90% coverage.

1. **Supplementary Figures**

**Figure S1.**

Comparison of AML- and B-ALL scores of B-ALL, AML and MPAL in 12 cases.

**Figure S2.**

Breakpoints of *BCR* and *ABL1* genes in each case.

**Figure S3.**

*MAP2K2-AC010132* fusion gene.

**Figure S4.**

Canonical pathways significantly enriched in MPAL.

**Figure S1.** Comparison of AML- and B-ALL scores of B-ALL, AML and MPAL in 12 cases.

^*^*P*=0.007, ^**^*P*=0.010.

A B


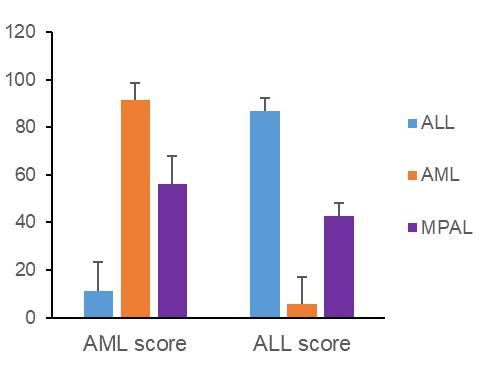

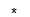

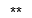


**Figure S2.** Breakpoints of *BCR* and *ABL1* genes in each case.


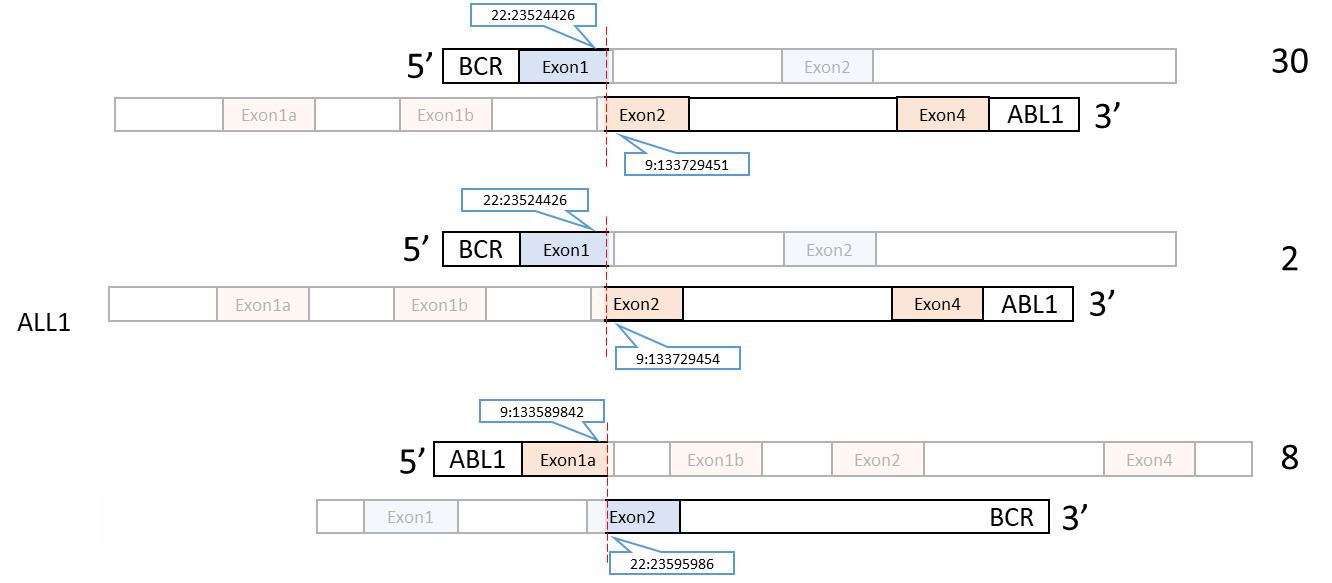


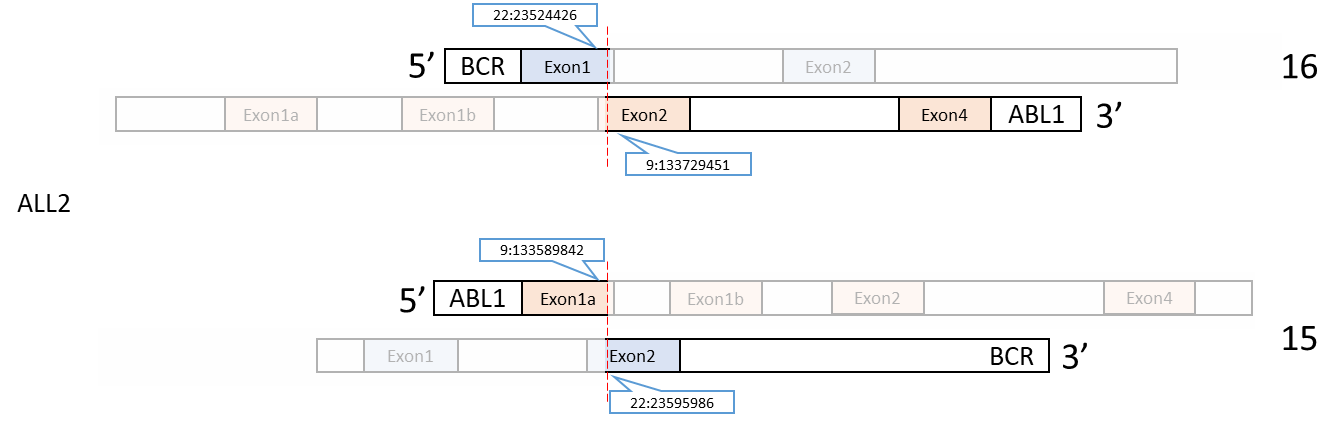


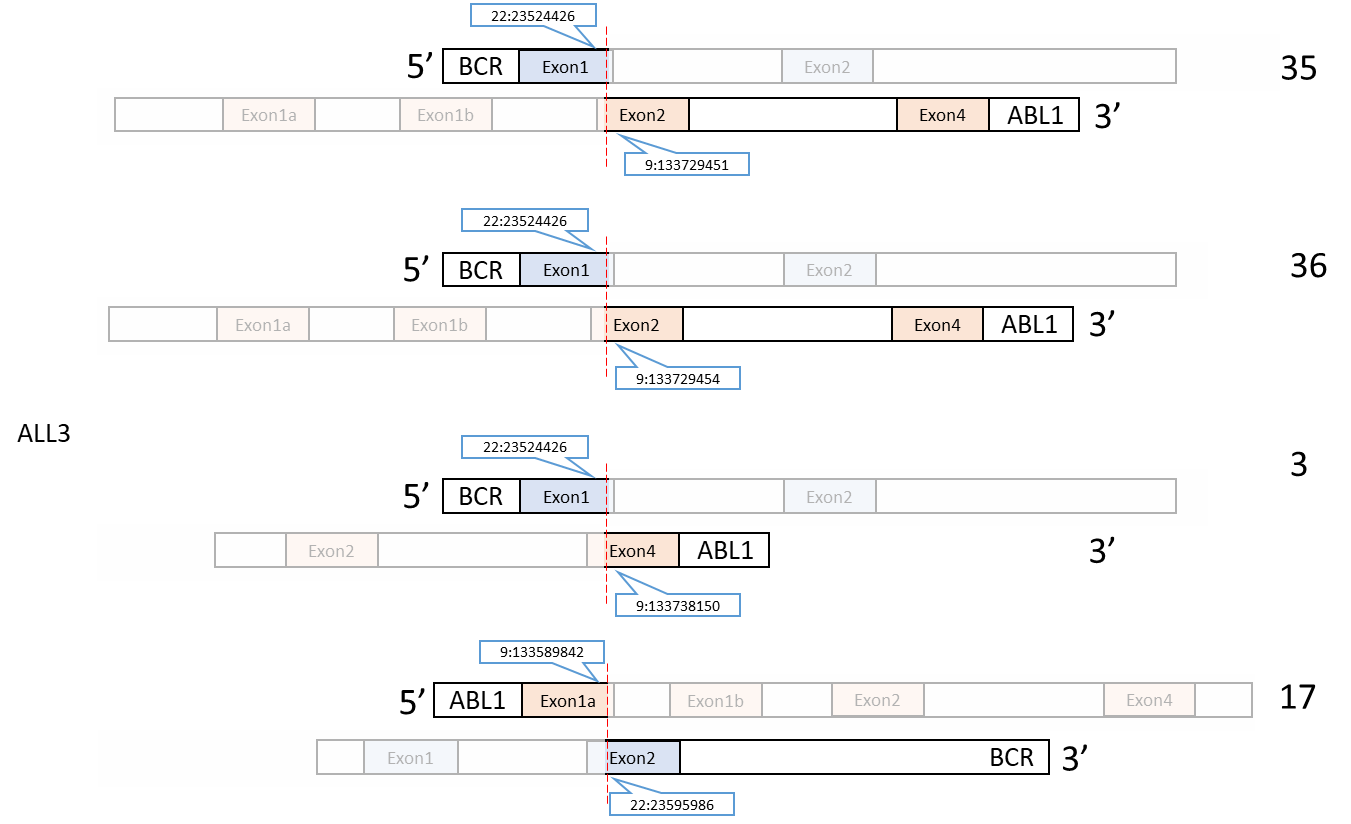


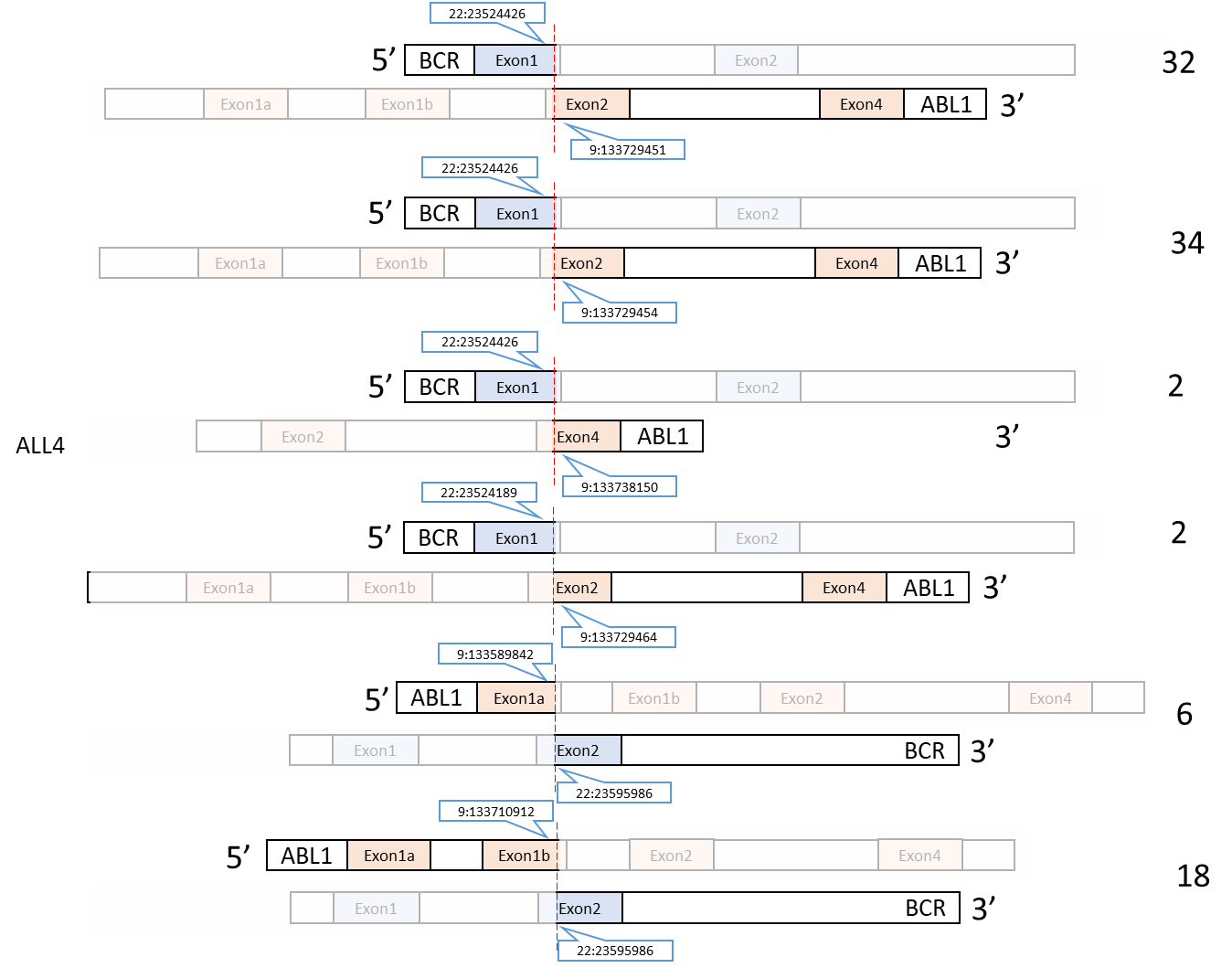


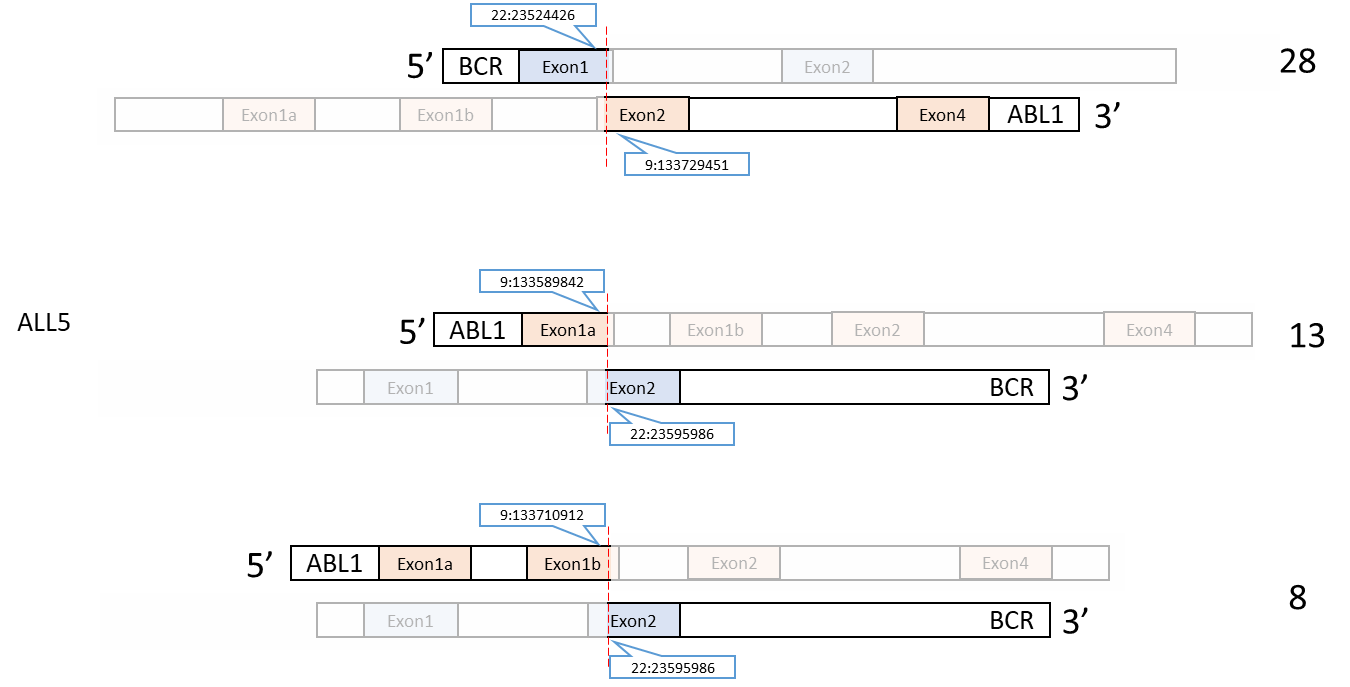


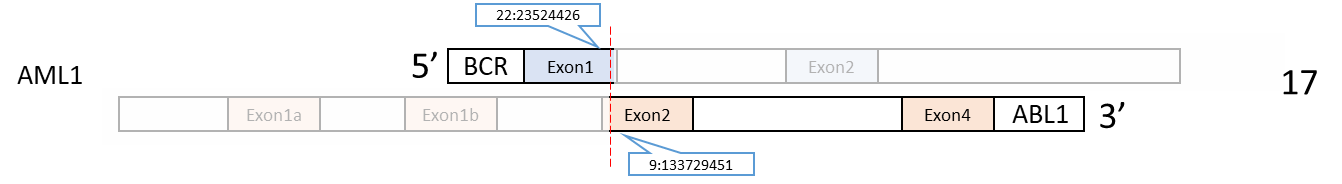


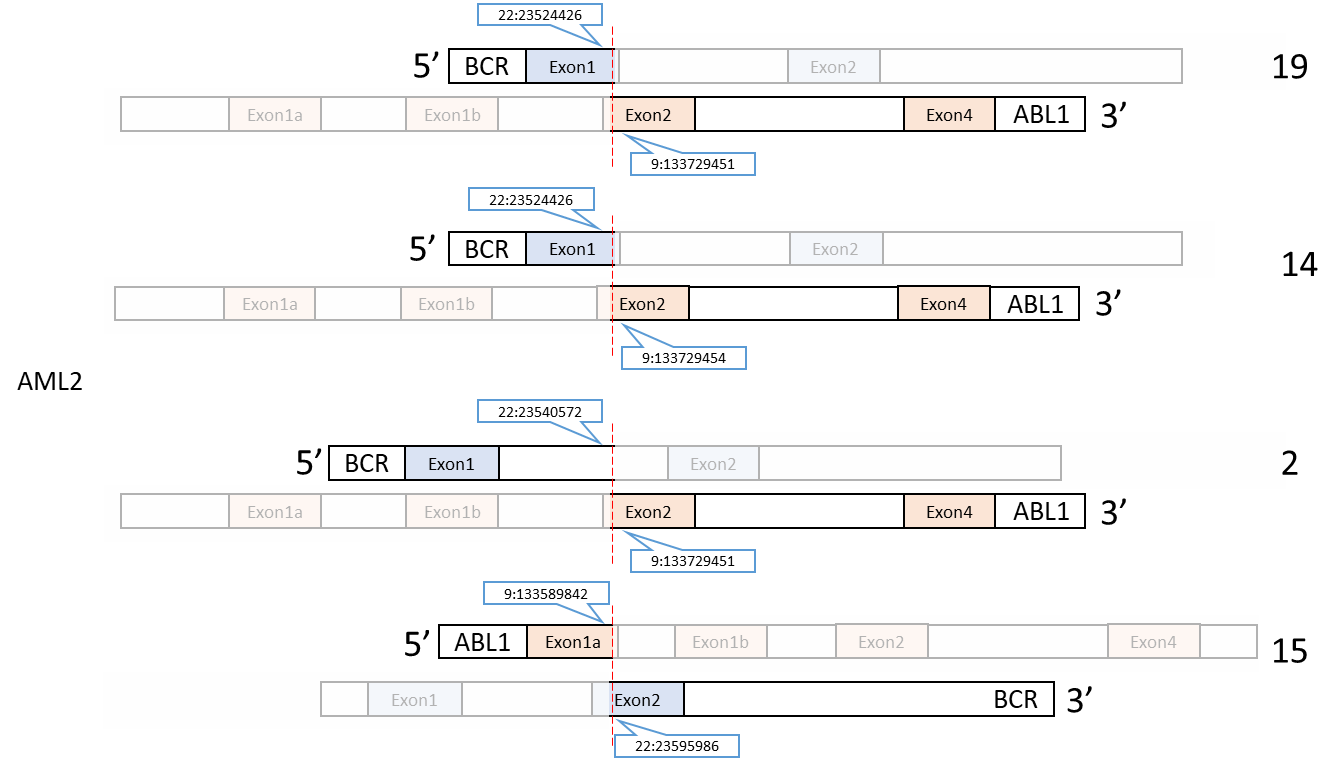


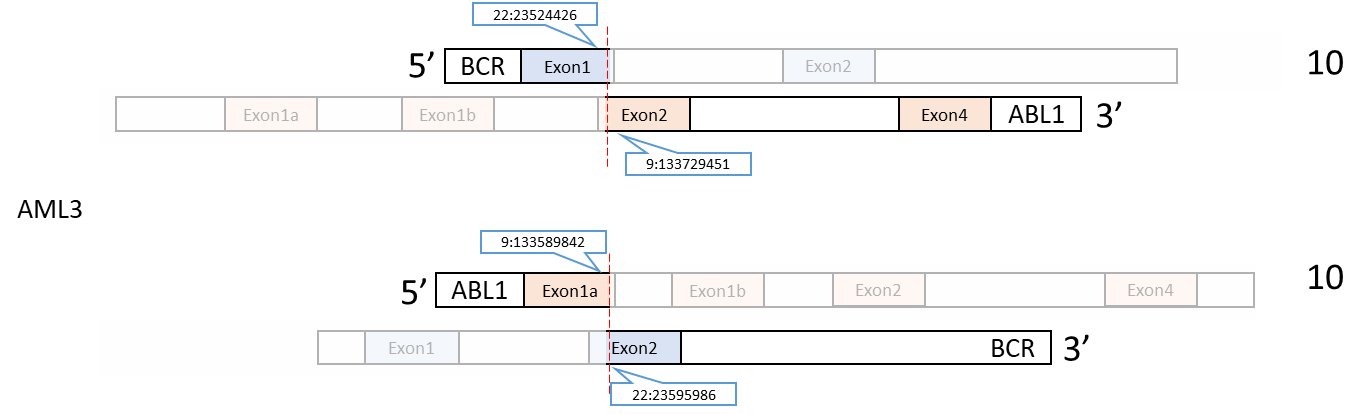


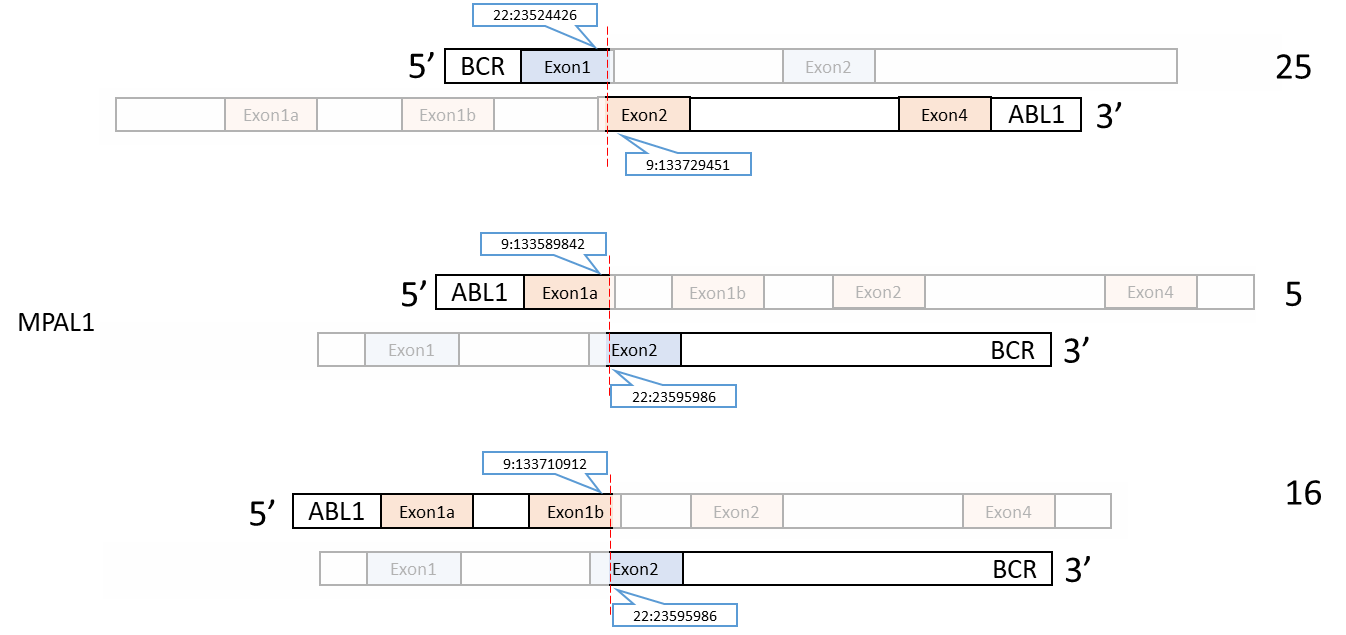


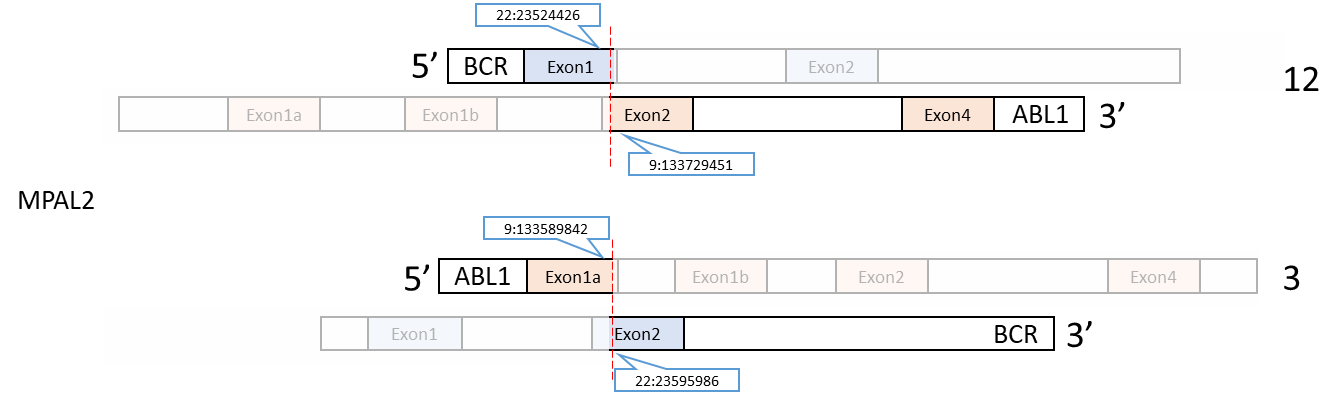


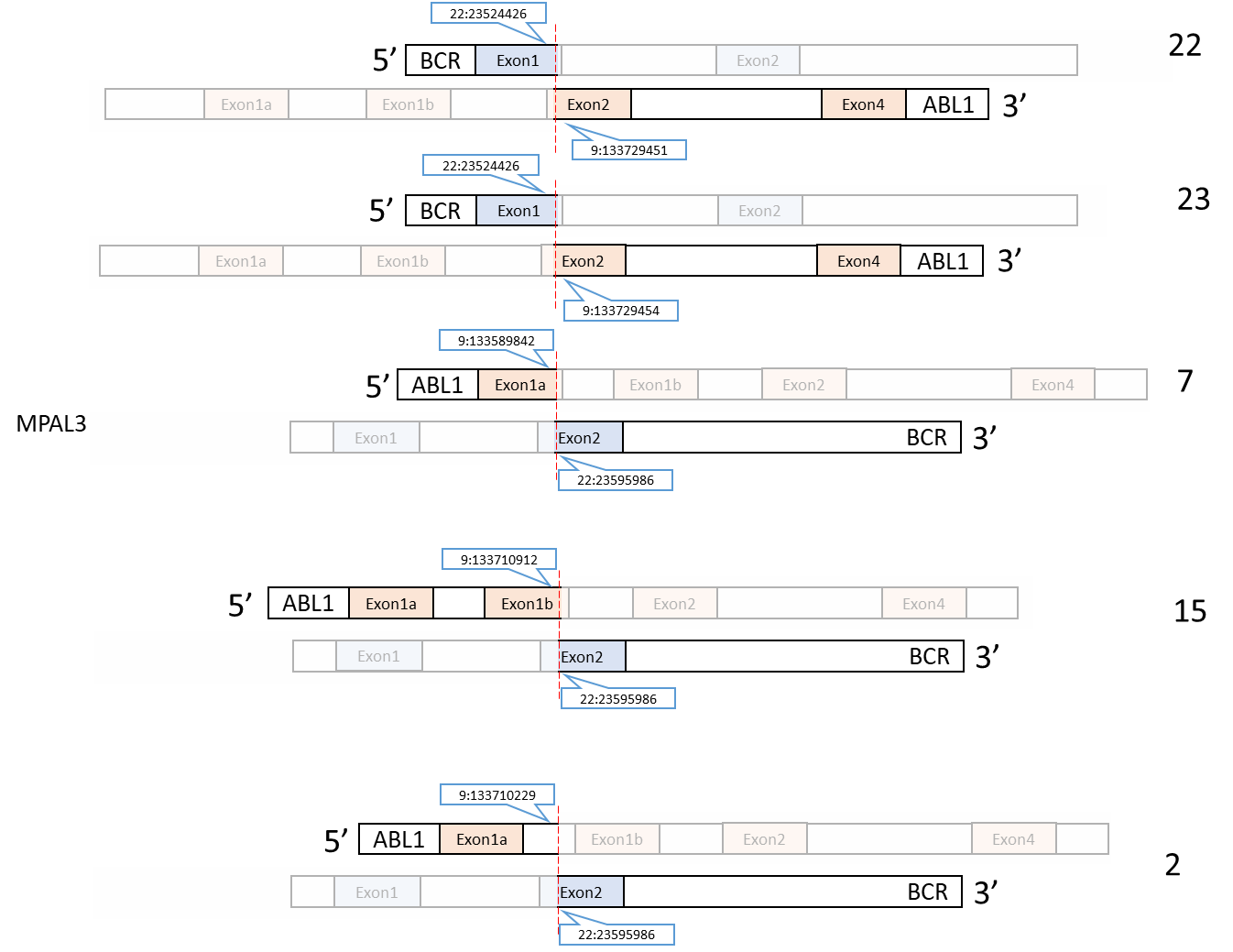


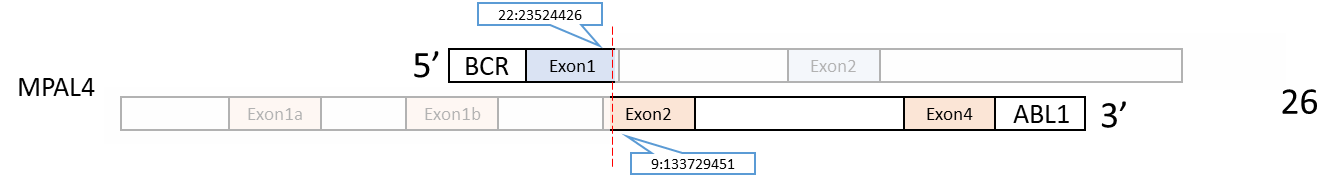


speech box – [chromosome : hg19 location]; The numbers on the right denote read counts of each fusion.

**Figure S3.** *MAP2K2-AC010132* fusion gene; sequence of the chimeric *MAP2K2-AC010132* gene between *AC010132* and exon10 of *MAP2K2*. Primer sequence of *MAP2K2*: GTG AAC GAG CCA CCT CCT AA and *AC010132.5*: CTT ACC ACA GGT CAG GAA ACC.


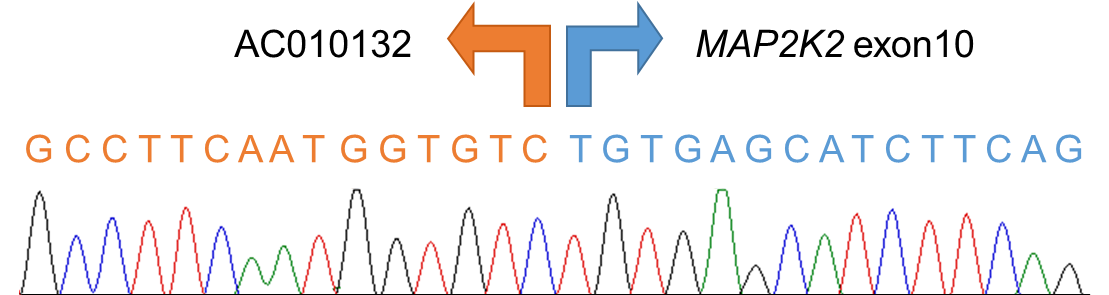


**Figure S4.**

4-1. Canonical pathways significantly enriched in mixed-phenotype acute leukemia (MPAL).

The z-score indicates predicted activation state of the canonical pathway. Orange color indicates a negative z-score and downregulation of the pathway in MPAL. Blue color indicates a positive z-score and upregulation of the pathway. The intensity of the bar graph color reflects the z-score. The ratio indicates the number of significantly expressed genes compared with the total number of genes associated with the canonical pathway. *Data compared with ALL, and others compared with AML.


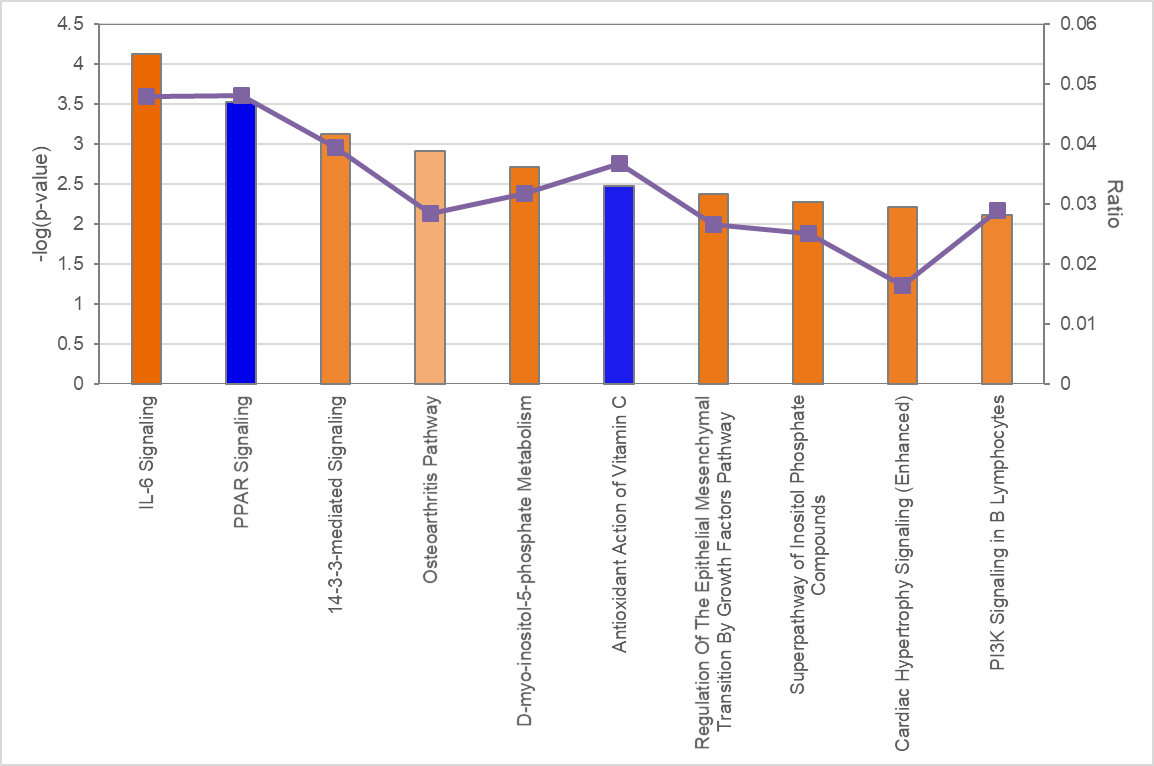


positive z-score

negative z-score

Ratio

*

4-2. IPA-based network

IPA-based network of mixed phenotype acute leukemia vs. acute myeloid leukemia (A) and acute lymphoblastic leukemia (B).

The solid lines indicate direct relationships, and dotted lines indicate indirect relationships between the pathways and molecules.


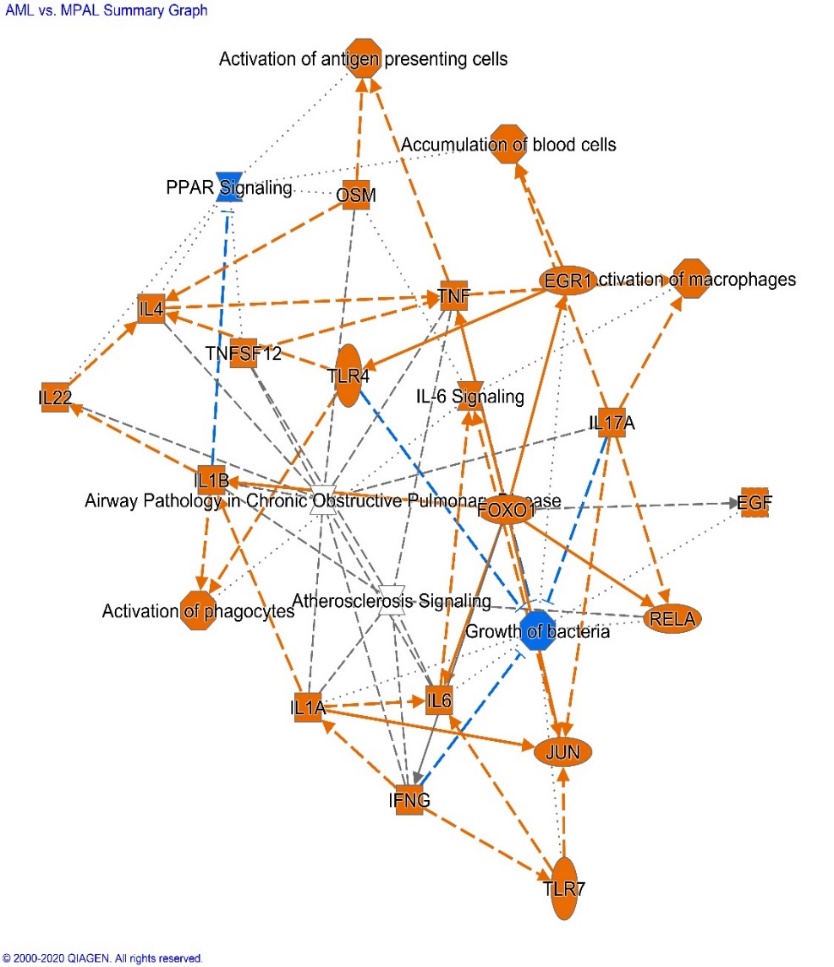

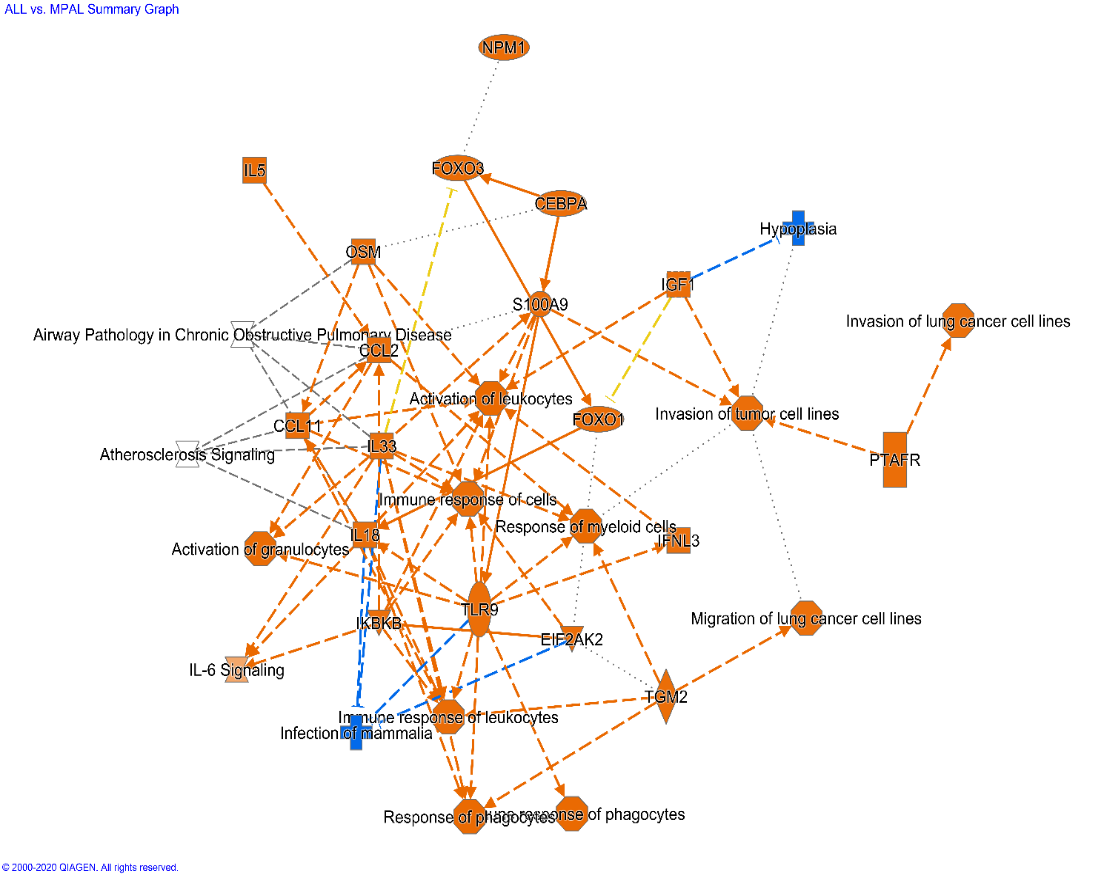


(A)

(B)

1. **Supplementary Tables**

**Table S1.**

List of AML- and B-ALL-specific genes and their cut-off FPKM level.

**Table S2.**

Gene Ontologies showing significant difference in each three group.

**Table S3.**

Gene sets with FDR less than 0.25 and with nominal p-value less than 0.01 in GSEA of AML and B-ALL.

**Table S4.**

Scoring system result in public expression data.

**Table S1.**

List of AML- and ALL-specific genes and their cut-off FPKM (Fragments Per Kilobase of exon per Million) level.

| Disease | Gene | FPKM |  | Disease | Gene | FPKM |
| --- | --- | --- | --- | --- | --- | --- |
| AML | *ACTN1* | 176 |  | ALL | *ABCD4* | 86 |
| AML | *ADCY7* | 118 |  | ALL | *ABHD17B* | 81 |
| AML | *ADIPOR1* | 94 |  | ALL | *AP1B1* | 114 |
| AML | *AGTRAP* | 83 |  | ALL | *ARHGEF7* | 100 |
| AML | *AHNAK* | 91 |  | ALL | *BAALC* | 94 |
| AML | *AHSP* | 117 |  | ALL | *BLNK* | 97 |
| AML | *ALAS2* | 116 |  | ALL | *BTNL9* | 83 |
| AML | *ALOX5* | 142 |  | ALL | *C12orf57* | 146 |
| AML | *ALOX5AP* | 110 |  | ALL | *CCND3* | 525 |
| AML | *AMICA1* | 95 |  | ALL | *CD19* | 235 |
| AML | *ANKRD44* | 169 |  | ALL | *CD22* | 127 |
| AML | *ANTXR2* | 95 |  | ALL | *CD34* | 377 |
| AML | *ANXA1* | 471 |  | ALL | *CD52* | 552 |
| AML | *ANXA2* | 214 |  | ALL | *CD69* | 350 |
| AML | *ANXA6* | 129 |  | ALL | *CD79A* | 154 |
| AML | *APLP2* | 390 |  | ALL | *CD79B* | 241 |
| AML | *APMAP* | 95 |  | ALL | *CD81* | 141 |
| AML | *APP* | 109 |  | ALL | *CD9* | 645 |
| AML | *ARAP1* | 117 |  | ALL | *CDK9* | 265 |
| AML | *ARL6IP5* | 123 |  | ALL | *CHD3* | 191 |
| AML | *ARPC1B* | 423 |  | ALL | *CHST15* | 214 |
| AML | *ARRB2* | 175 |  | ALL | *CLEC11A* | 91 |
| AML | *ASAH1* | 179 |  | ALL | *CSNK1E* | 81 |
| AML | *ATG16L2* | 231 |  | ALL | *CTGF* | 188 |
| AML | *AZU1* | 132 |  | ALL | *DDIT4* | 156 |
| AML | *BIN2* | 97 |  | ALL | *DDR1* | 103 |
| AML | *BST2* | 104 |  | ALL | *DDX11* | 96 |
| AML | *BZRAP1* | 128 |  | ALL | *DENND3* | 496 |
| AML | *C10orf54* | 200 |  | ALL | *DNTT* | 828 |
| AML | *C3AR1* | 128 |  | ALL | *DPEP1* | 125 |
| AML | *CA1* | 165 |  | ALL | *ECM1* | 174 |
| AML | *CD14* | 204 |  | ALL | *EDEM1* | 188 |
| AML | *CD33* | 87 |  | ALL | *ELK3* | 100 |
| AML | *CD36* | 200 |  | ALL | *EMP1* | 103 |
| AML | *CD44* | 206 |  | ALL | *ENG* | 113 |
| AML | *CD63* | 149 |  | ALL | *ERG* | 105 |
| AML | *CD68* | 196 |  | ALL | *ERGIC1* | 149 |
| Disease | Gene | FPKM |  | Disease | Gene | FPKM |
| AML | *CDKN1A* | 272 |  | ALL | *EVL* | 132 |
| AML | *CECR1* | 188 |  | ALL | *FAIM3* | 100 |
| AML | *CFLAR* | 94 |  | ALL | *FBXW7* | 110 |
| AML | *CFP* | 96 |  | ALL | *FHL1* | 173 |
| AML | *CITED2* | 137 |  | ALL | *FPGS* | 174 |
| AML | *CLC* | 513 |  | ALL | *GAB1* | 178 |
| AML | *CLEC12A* | 245 |  | ALL | *GATS* | 86 |
| AML | *CLEC5A* | 83 |  | ALL | *GBP4* | 83 |
| AML | *CORO1A* | 380 |  | ALL | *GPR110* | 121 |
| AML | *COTL1* | 199 |  | ALL | *GSN* | 307 |
| AML | *CPNE3* | 277 |  | ALL | *H1F0* | 219 |
| AML | *CSF1R* | 84 |  | ALL | *HDAC7* | 122 |
| AML | *CSF3R* | 319 |  | ALL | *HIST1H1C* | 85 |
| AML | *CST7* | 171 |  | ALL | *HIST1H2BK* | 145 |
| AML | *CSTA* | 171 |  | ALL | *HLA-DOA* | 119 |
| AML | *CTSA* | 85 |  | ALL | *HLA-DQA1* | 149 |
| AML | *CTSB* | 204 |  | ALL | *HLA-F* | 201 |
| AML | *CTSC* | 94 |  | ALL | *HPS4* | 238 |
| AML | *CTSD* | 312 |  | ALL | *HSPB1* | 114 |
| AML | *CTSG* | 195 |  | ALL | *IFI16* | 158 |
| AML | *CTSS* | 542 |  | ALL | *ITGA6* | 131 |
| AML | *CTSZ* | 113 |  | ALL | *JUP* | 222 |
| AML | *CYBB* | 237 |  | ALL | *LEF1* | 484 |
| AML | *DEFA3* | 473 |  | ALL | *LIMD1* | 118 |
| AML | *DEFA4* | 83 |  | ALL | *LIMD1-AS1* | 98 |
| AML | *DOCK2* | 84 |  | ALL | *LOC101144319* | 164 |
| AML | *EFHD2* | 85 |  | ALL | *LPAR6* | 149 |
| AML | *ELANE* | 215 |  | ALL | *LRMP* | 259 |
| AML | *ELMO1* | 101 |  | ALL | *MARCKSL1* | 103 |
| AML | *EMB* | 253 |  | ALL | *MDM2* | 208 |
| AML | *EPX* | 229 |  | ALL | *MEF2C* | 279 |
| AML | *EVI2B* | 198 |  | ALL | *MLXIP* | 110 |
| AML | *FAM101B* | 83 |  | ALL | *MME(CD10)* | 256 |
| AML | *FBXL5* | 107 |  | ALL | *MS4A1(CD20)* | 171 |
| AML | *FCER1G* | 215 |  | ALL | *MSH6* | 127 |
| AML | *FCGR2A* | 89 |  | ALL | *MSI2* | 116 |
| AML | *FCN1* | 611 |  | ALL | *MZB1* | 332 |
| AML | *FGL2* | 218 |  | ALL | *NARFL* | 111 |
| AML | *FGR* | 218 |  | ALL | *NPIPB3* | 149 |
| AML | *FLNA* | 357 |  | ALL | *PARP1* | 255 |
| AML | *FLOT2* | 118 |  | ALL | *PBXIP1* | 146 |
| Disease | Gene | FPKM |  | Disease | Gene | FPKM |
| AML | *FNDC3B* | 96 |  | ALL | *PPIP5K2* | 109 |
| AML | *FOS* | 501 |  | ALL | *PRDX1* | 540 |
| AML | *FOSL2* | 95 |  | ALL | *PSMA6* | 430 |
| AML | *FTL* | 942 |  | ALL | *PTPN18* | 162 |
| AML | *G6PD* | 123 |  | ALL | *PXDN* | 213 |
| AML | *GCA* | 134 |  | ALL | *RAG1* | 91 |
| AML | *GLIPR1* | 138 |  | ALL | *RAG2* | 86 |
| AML | *GLIPR2* | 86 |  | ALL | *RCBTB1* | 94 |
| AML | *GLUL* | 371 |  | ALL | *RFX5* | 140 |
| AML | *GPI* | 239 |  | ALL | *S100A13* | 129 |
| AML | *GRB2* | 210 |  | ALL | *S100A16* | 297 |
| AML | *GRN* | 444 |  | ALL | *SEPT9* | 348 |
| AML | *HBA1* | 581 |  | ALL | *SH2D3C* | 117 |
| AML | *HBA2* | 915 |  | ALL | *SH3BP5* | 85 |
| AML | *HBB* | 15038 |  | ALL | *SLC2A5* | 145 |
| AML | *HBD* | 235 |  | ALL | *SLC38A1* | 175 |
| AML | *HBG2* | 572 |  | ALL | *SLC44A2* | 101 |
| AML | *HCST* | 109 |  | ALL | *SNX8* | 112 |
| AML | *HDC* | 92 |  | ALL | *SOCS2* | 415 |
| AML | *HGF* | 135 |  | ALL | *SORBS3* | 133 |
| AML | *HK3* | 81 |  | ALL | *SPATS2L* | 111 |
| AML | *HSD17B11* | 82 |  | ALL | *SPTBN1* | 92 |
| AML | *HSH2D* | 116 |  | ALL | *SSBP2* | 202 |
| AML | *IFI30* | 683 |  | ALL | *STAG3* | 288 |
| AML | *IFI44* | 85 |  | ALL | *TAPT1* | 82 |
| AML | *IFI44L* | 81 |  | ALL | *TBCD* | 113 |
| AML | *IFI6* | 141 |  | ALL | *TCF12* | 91 |
| AML | *IGHA1* | 490 |  | ALL | *TCF3* | 234 |
| AML | *IGHA2* | 126 |  | ALL | *TCF4* | 84 |
| AML | *IGHG1* | 455 |  | ALL | *TCL1A* | 562 |
| AML | *IGHG2* | 100 |  | ALL | *TOP2B* | 256 |
| AML | *IGHG4* | 100 |  | ALL | *TP53INP1* | 106 |
| AML | *IGHV3-7* | 156 |  | ALL | *TSPAN7* | 129 |
| AML | *IGKC* | 488 |  | ALL | *UBASH3B* | 112 |
| AML | *IGKV1-5* | 94 |  | ALL | *VPREB1* | 239 |
| AML | *IGKV3-11* | 147 |  | ALL | *XBP1* | 509 |
| AML | *IGKV3-20* | 179 |  | ALL | *YBX3* | 405 |
| AML | *IGLC2* | 137 |  | ALL | *ZCCHC7* | 211 |
| AML | *IGLC3* | 113 |  | ALL | *ZFP36L1* | 108 |
| AML | *IL10RA* | 88 |  |  |  |  |
| AML | *IRF7* | 96 |  |  |  |  |
| Disease | Gene | FPKM |  | Disease | Gene | FPKM |
| AML | *IRF9* | 89 |  |  |  |  |
| AML | *ITGAL* | 119 |  |  |  |  |
| AML | *ITGAM* | 216 |  |  |  |  |
| AML | *ITGAX* | 84 |  |  |  |  |
| AML | *ITGB2* | 379 |  |  |  |  |
| AML | *ITPK1* | 92 |  |  |  |  |
| AML | *JUNB* | 81 |  |  |  |  |
| AML | *KCNAB2* | 134 |  |  |  |  |
| AML | *KIAA0930* | 87 |  |  |  |  |
| AML | *LAMP2* | 107 |  |  |  |  |
| AML | *LCP1* | 595 |  |  |  |  |
| AML | *LGALS1* | 516 |  |  |  |  |
| AML | *LILRB2* | 89 |  |  |  |  |
| AML | *LSP1* | 108 |  |  |  |  |
| AML | *LST1* | 172 |  |  |  |  |
| AML | *LTA4H* | 143 |  |  |  |  |
| AML | *LTF* | 487 |  |  |  |  |
| AML | *LY6E* | 113 |  |  |  |  |
| AML | *LYN* | 83 |  |  |  |  |
| AML | *LYST* | 114 |  |  |  |  |
| AML | *LYZ* | 6308 |  |  |  |  |
| AML | *MAN2B1* | 105 |  |  |  |  |
| AML | *MAPK14* | 100 |  |  |  |  |
| AML | *MBOAT7* | 150 |  |  |  |  |
| AML | *MCL1* | 318 |  |  |  |  |
| AML | *MFSD1* | 169 |  |  |  |  |
| AML | *MMP8* | 84 |  |  |  |  |
| AML | *MNDA* | 251 |  |  |  |  |
| AML | *MPEG1* | 154 |  |  |  |  |
| AML | *MPO* | 2090 |  |  |  |  |
| AML | *MRPL33* | 126 |  |  |  |  |
| AML | *MS4A3* | 263 |  |  |  |  |
| AML | *MS4A6A* | 198 |  |  |  |  |
| AML | *MX1* | 107 |  |  |  |  |
| AML | *MYADM* | 115 |  |  |  |  |
| AML | *MYC* | 92 |  |  |  |  |
| AML | *MYD88* | 119 |  |  |  |  |
| AML | *MYO1F* | 243 |  |  |  |  |
| AML | *MYO1G* | 105 |  |  |  |  |
| AML | *NADK* | 190 |  |  |  |  |
| AML | *NAGK* | 89 |  |  |  |  |
| Disease | Gene | FPKM |  | Disease | Gene | FPKM |
| AML | *NCF2* | 138 |  |  |  |  |
| AML | *NCOA4* | 316 |  |  |  |  |
| AML | *NFE2* | 103 |  |  |  |  |
| AML | *NUCB2* | 116 |  |  |  |  |
| AML | *P4HB* | 513 |  |  |  |  |
| AML | *PADI4* | 111 |  |  |  |  |
| AML | *PAK1* | 107 |  |  |  |  |
| AML | *PGD* | 314 |  |  |  |  |
| AML | *PGLYRP1* | 93 |  |  |  |  |
| AML | *PLAC8* | 190 |  |  |  |  |
| AML | *PLCB2* | 177 |  |  |  |  |
| AML | *PLD3* | 96 |  |  |  |  |
| AML | *PLEK* | 150 |  |  |  |  |
| AML | *PLP2* | 81 |  |  |  |  |
| AML | *PLSCR1* | 114 |  |  |  |  |
| AML | *PLXNA3* | 205 |  |  |  |  |
| AML | *PRAM1* | 110 |  |  |  |  |
| AML | *PRG2* | 576 |  |  |  |  |
| AML | *PRKCD* | 89 |  |  |  |  |
| AML | *PRTN3* | 174 |  |  |  |  |
| AML | *PSAP* | 684 |  |  |  |  |
| AML | *PTPN6* | 204 |  |  |  |  |
| AML | *PTPRC* | 256 |  |  |  |  |
| AML | *PYGL* | 93 |  |  |  |  |
| AML | *RAB27A* | 133 |  |  |  |  |
| AML | *RAB31* | 86 |  |  |  |  |
| AML | *RAB7A* | 244 |  |  |  |  |
| AML | *RAC2* | 390 |  |  |  |  |
| AML | *RGS2* | 122 |  |  |  |  |
| AML | *RHOG* | 109 |  |  |  |  |
| AML | *RNASE2* | 760 |  |  |  |  |
| AML | *RNASE3* | 154 |  |  |  |  |
| AML | *RTN3* | 152 |  |  |  |  |
| AML | *S100A10* | 97 |  |  |  |  |
| AML | *S100A11* | 195 |  |  |  |  |
| AML | *S100A12* | 164 |  |  |  |  |
| AML | *S100A4* | 560 |  |  |  |  |
| AML | *S100A6* | 308 |  |  |  |  |
| AML | *S100A8* | 3027 |  |  |  |  |
| AML | *S100A9* | 4073 |  |  |  |  |
| AML | *SAMHD1* | 178 |  |  |  |  |
| Disease | Gene | FPKM |  | Disease | Gene | FPKM |
| AML | *SAT1* | 341 |  |  |  |  |
| AML | *SCPEP1* | 120 |  |  |  |  |
| AML | *SDCBP* | 166 |  |  |  |  |
| AML | *SEC11A* | 85 |  |  |  |  |
| AML | *SELL* | 443 |  |  |  |  |
| AML | *SERPINA1* | 158 |  |  |  |  |
| AML | *SERPINB1* | 331 |  |  |  |  |
| AML | *SIRPB1* | 104 |  |  |  |  |
| AML | *SLC11A1* | 159 |  |  |  |  |
| AML | *SLC16A3* | 132 |  |  |  |  |
| AML | *SLC25A37* | 143 |  |  |  |  |
| AML | *SLC2A3* | 82 |  |  |  |  |
| AML | *SLC4A1* | 116 |  |  |  |  |
| AML | *SLC7A7* | 83 |  |  |  |  |
| AML | *SORL1* | 80 |  |  |  |  |
| AML | *SPI1* | 198 |  |  |  |  |
| AML | *SPN* | 100 |  |  |  |  |
| AML | *SRGN* | 4263 |  |  |  |  |
| AML | *STAT1* | 114 |  |  |  |  |
| AML | *STAT2* | 145 |  |  |  |  |
| AML | *STOM* | 155 |  |  |  |  |
| AML | *STXBP2* | 114 |  |  |  |  |
| AML | *SULF2* | 86 |  |  |  |  |
| AML | *SVIL* | 258 |  |  |  |  |
| AML | *TAGLN2* | 339 |  |  |  |  |
| AML | *TALDO1* | 163 |  |  |  |  |
| AML | *TKT* | 504 |  |  |  |  |
| AML | *TMBIM1* | 105 |  |  |  |  |
| AML | *TMEM30A* | 102 |  |  |  |  |
| AML | *TNFAIP2* | 174 |  |  |  |  |
| AML | *TNFAIP8* | 103 |  |  |  |  |
| AML | *TNFRSF1B* | 123 |  |  |  |  |
| AML | *TPP1* | 229 |  |  |  |  |
| AML | *TRAF3IP3* | 84 |  |  |  |  |
| AML | *TSPO* | 136 |  |  |  |  |
| AML | *TYROBP* | 323 |  |  |  |  |
| AML | *UNC13D* | 127 |  |  |  |  |
| AML | *VAMP8* | 127 |  |  |  |  |
| AML | *VASP* | 91 |  |  |  |  |
| AML | *VAT1* | 122 |  |  |  |  |
| AML | *VCAN* | 89 |  |  |  |  |
| Disease | Gene | FPKM |  | Disease | Gene | FPKM |
| AML | *VIM* | 190 |  |  |  |  |
| AML | *VMP1* | 133 |  |  |  |  |
| AML | *WARS* | 109 |  |  |  |  |
| AML | *WAS* | 107 |  |  |  |  |
| AML | *XAF1* | 237 |  |  |  |  |
| AML | *YWHAH* | 125 |  |  |  |  |
| AML | *ZEB2* | 88 |  |  |  |  |
| AML | *ZFP36* | 189 |  |  |  |  |
| AML | *ZYX* | 273 |  |  |  |  |

Orange-colored genes: genes coding proteins used in phenotyping study of AML diagnosis. Blue-colored genes: genes coding proteins used in phenotyping analysis of B-ALL diagnosis.

**Table S2**.

Gene Ontologies showing significant differences in each three group.

| GO_ID | Category | Name | *P*  (B-ALL vs AML) | *P*  (B-ALL vs MPAL) | *P*  (AML vs  MPAL) |
| --- | --- | --- | --- | --- | --- |
| GO:0000165 | biological_process | MAPK cascade | 0 | 0.000211 | 0.000275 |
| GO:0001525 | biological_process | angiogenesis | 0 | 0.000009 | 0.000002 |
| GO:0001568 | biological_process | blood vessel development | 0 | 0 | 0.000017 |
| GO:0001775 | biological_process | cell activation | 0 | 0 | 0 |
| GO:0001816 | biological_process | cytokine production | 0 | 0 | 0 |
| GO:0001817 | biological_process | regulation of cytokine production | 0 | 0 | 0 |
| GO:0001818 | biological_process | negative regulation of cytokine production | 0 | 0.00016 | 0.000019 |
| GO:0001819 | biological_process | positive regulation of cytokine production | 0 | 0.000074 | 0 |
| GO:0001932 | biological_process | regulation of protein phosphorylation | 0 | 0.000371 | 0.000004 |
| GO:0001934 | biological_process | positive regulation of protein phosphorylation | 0 | 0.000394 | 0.000001 |
| GO:0001944 | biological_process | vasculature development | 0 | 0 | 0.000042 |
| GO:0002237 | biological_process | response to molecule of bacterial origin | 0 | 0 | 0.000003 |
| GO:0002250 | biological_process | adaptive immune response | 0 | 0.000175 | 0.000272 |
| GO:0002253 | biological_process | activation of immune response | 0 | 0 | 0.00024 |
| GO:0002376 | biological_process | immune system process | 0 | 0 | 0 |
| GO:0002443 | biological_process | leukocyte mediated immunity | 0 | 0.000033 | 0.000331 |
| GO:0002460 | biological_process | adaptive immune response based on somatic recombination of immune receptors built from immunoglobulin superfamily domains | 0 | 0.000233 | 0.000076 |
| GO:0002520 | biological_process | immune system development | 0 | 0.000008 | 0.000004 |
| GO_ID | Category | Name | *P*  (B-ALL vs AML) | *P*  (B-ALL vs MPAL) | *P*  (AML vs  MPAL) |
| GO:0002521 | biological_process | leukocyte differentiation | 0 | 0 | 0.000004 |
| GO:0002573 | biological_process | myeloid leukocyte differentiation | 0.000823 | 0.000409 | 0.000118 |
| GO:0002682 | biological_process | regulation of immune system process | 0 | 0 | 0 |
| GO:0002684 | biological_process | positive regulation of immune system process | 0 | 0 | 0.000001 |
| GO:0004872 | molecular_function | receptor activity | 0 | 0 | 0.000266 |
| GO:0005102 | molecular_function | receptor binding | 0 | 0.000003 | 0.000012 |
| GO:0005515 | molecular_function | protein binding | 0 | 0.000013 | 0.000006 |
| GO:0005576 | cellular_component | extracellular region | 0 | 0 | 0 |
| GO:0005615 | cellular_component | extracellular space | 0 | 0 | 0 |
| GO:0005886 | cellular_component | plasma membrane | 0 | 0 | 0 |
| GO:0005887 | cellular_component | integral to plasma membrane | 0 | 0 | 0.000015 |
| GO:0006796 | biological_process | phosphate-containing compound metabolic process | 0 | 0.000259 | 0.000507 |
| GO:0006873 | biological_process | cellular ion homeostasis | 0.000093 | 0.000959 | 0.000401 |
| GO:0006915 | biological_process | apoptotic process | 0 | 0 | 0 |
| GO:0006928 | biological_process | cellular component movement | 0 | 0 | 0 |
| GO:0006935 | biological_process | chemotaxis | 0 | 0 | 0.000054 |
| GO:0006950 | biological_process | response to stress | 0 | 0 | 0 |
| GO:0006952 | biological_process | defense response | 0 | 0 | 0 |
| GO:0006954 | biological_process | inflammatory response | 0 | 0 | 0 |
| GO:0006955 | biological_process | immune response | 0 | 0 | 0 |
| GO:0006979 | biological_process | response to oxidative stress | 0.000018 | 0 | 0.000135 |
| GO:0007154 | biological_process | cell communication | 0 | 0 | 0 |
| GO_ID | Category | Name | *P*  (B-ALL vs AML) | *P*  (B-ALL vs MPAL) | *P*  (AML vs  MPAL) |
| GO:0007155 | biological_process | cell adhesion | 0 | 0 | 0.000604 |
| GO:0007165 | biological_process | signal transduction | 0 | 0 | 0 |
| GO:0007166 | biological_process | cell surface receptor signaling pathway | 0 | 0 | 0 |
| GO:0007167 | biological_process | enzyme linked receptor protein signaling pathway | 0 | 0.000087 | 0.00007 |
| GO:0007169 | biological_process | transmembrane receptor protein tyrosine kinase signaling pathway | 0 | 0.000608 | 0.000893 |
| GO:0007243 | biological_process | intracellular protein kinase cascade | 0 | 0.000047 | 0.000012 |
| GO:0007275 | biological_process | multicellular organismal development | 0 | 0 | 0.000002 |
| GO:0008219 | biological_process | cell death | 0 | 0 | 0.000001 |
| GO:0008283 | biological_process | cell proliferation | 0 | 0 | 0.00001 |
| GO:0008284 | biological_process | positive regulation of cell proliferation | 0.000023 | 0.000124 | 0.000365 |
| GO:0008289 | molecular_function | lipid binding | 0 | 0.000602 | 0.000018 |
| GO:0009306 | biological_process | protein secretion | 0 | 0 | 0.000019 |
| GO:0009605 | biological_process | response to external stimulus | 0 | 0 | 0 |
| GO:0009607 | biological_process | response to biotic stimulus | 0 | 0 | 0 |
| GO:0009611 | biological_process | response to wounding | 0 | 0 | 0 |
| GO:0009617 | biological_process | response to bacterium | 0 | 0 | 0 |
| GO:0009653 | biological_process | anatomical structure morphogenesis | 0 | 0 | 0.000006 |
| GO:0009893 | biological_process | positive regulation of metabolic process | 0 | 0.000576 | 0.000123 |
| GO:0009897 | cellular_component | external side of plasma membrane | 0 | 0 | 0.000002 |
| GO:0009966 | biological_process | regulation of signal transduction | 0 | 0 | 0 |
| GO:0009967 | biological_process | positive regulation of signal transduction | 0 | 0.000204 | 0.00005 |
| GO_ID | Category | Name | *P*  (B-ALL vs AML) | *P*  (B-ALL vs MPAL) | *P*  (AML vs  MPAL) |
| GO:0009986 | cellular_component | cell surface | 0 | 0 | 0.00006 |
| GO:0010033 | biological_process | response to organic substance | 0 | 0 | 0 |
| GO:0010627 | biological_process | regulation of intracellular protein kinase cascade | 0 | 0.000075 | 0.00001 |
| GO:0010646 | biological_process | regulation of cell communication | 0 | 0.000004 | 0 |
| GO:0010740 | biological_process | positive regulation of intracellular protein kinase cascade | 0 | 0.000534 | 0.000486 |
| GO:0010941 | biological_process | regulation of cell death | 0 | 0.000002 | 0 |
| GO:0012501 | biological_process | programmed cell death | 0 | 0 | 0 |
| GO:0016265 | biological_process | death | 0 | 0 | 0.000001 |
| GO:0016477 | biological_process | cell migration | 0 | 0 | 0 |
| GO:0019838 | molecular_function | growth factor binding | 0 | 0.000003 | 0.00005 |
| GO:0023014 | biological_process | signal transduction by phosphorylation | 0 | 0.000141 | 0.000318 |
| GO:0023051 | biological_process | regulation of signaling | 0 | 0.000003 | 0 |
| GO:0023052 | biological_process | signaling | 0 | 0 | 0 |
| GO:0030097 | biological_process | hemopoiesis | 0 | 0 | 0.000001 |
| GO:0030154 | biological_process | cell differentiation | 0 | 0 | 0.000005 |
| GO:0030198 | biological_process | extracellular matrix organization | 0 | 0 | 0.000689 |
| GO:0030225 | biological_process | macrophage differentiation | 0.000719 | 0.000042 | 0.000313 |
| GO:0030334 | biological_process | regulation of cell migration | 0 | 0.000008 | 0 |
| GO:0030335 | biological_process | positive regulation of cell migration | 0 | 0.000126 | 0 |
| GO:0031226 | cellular_component | intrinsic to plasma membrane | 0 | 0 | 0.00002 |
| GO_ID | Category | Name | *P*  (B-ALL vs AML) | *P*  (B-ALL vs MPAL) | *P*  (AML vs  MPAL) |
| GO:0032270 | biological_process | positive regulation of cellular protein metabolic process | 0.000001 | 0.00076 | 0.000134 |
| GO:0032496 | biological_process | response to lipopolysaccharide | 0 | 0 | 0.000008 |
| GO:0032502 | biological_process | developmental process | 0 | 0 | 0.000001 |
| GO:0032651 | biological_process | regulation of interleukin-1 beta production | 0.000017 | 0.000833 | 0.000622 |
| GO:0032674 | biological_process | regulation of interleukin-5 production | 0.000835 | 0.000941 | 0.000911 |
| GO:0032680 | biological_process | regulation of tumor necrosis factor production | 0 | 0.000003 | 0.000083 |
| GO:0032940 | biological_process | secretion by cell | 0 | 0.000002 | 0.000075 |
| GO:0033674 | biological_process | positive regulation of kinase activity | 0 | 0.000478 | 0.00001 |
| GO:0033993 | biological_process | response to lipid | 0 | 0.000002 | 0.00003 |
| GO:0034097 | biological_process | response to cytokine | 0 | 0.000102 | 0 |
| GO:0034599 | biological_process | cellular response to oxidative stress | 0.000262 | 0 | 0.000283 |
| GO:0035556 | biological_process | intracellular signal transduction | 0 | 0 | 0.000001 |
| GO:0040011 | biological_process | locomotion | 0 | 0 | 0 |
| GO:0040012 | biological_process | regulation of locomotion | 0 | 0.000001 | 0 |
| GO:0040017 | biological_process | positive regulation of locomotion | 0 | 0.000125 | 0.000001 |
| GO:0042035 | biological_process | regulation of cytokine biosynthetic process | 0.000045 | 0.000352 | 0.00062 |
| GO:0042089 | biological_process | cytokine biosynthetic process | 0.000003 | 0.000091 | 0.00029 |
| GO:0042107 | biological_process | cytokine metabolic process | 0.000001 | 0.000124 | 0.000351 |
| GO:0042127 | biological_process | regulation of cell proliferation | 0 | 0 | 0.000019 |
| GO:0042221 | biological_process | response to chemical | 0 | 0 | 0 |
| GO:0042325 | biological_process | regulation of phosphorylation | 0 | 0.000196 | 0.000003 |
| GO_ID | Category | Name | *P*  (B-ALL vs AML) | *P*  (B-ALL vs MPAL) | *P*  (AML vs  MPAL) |
| GO:0042327 | biological_process | positive regulation of phosphorylation | 0 | 0.000238 | 0.000004 |
| GO:0042742 | biological_process | defense response to bacterium | 0 | 0 | 0.000546 |
| GO:0042981 | biological_process | regulation of apoptotic process | 0 | 0.000004 | 0 |
| GO:0043062 | biological_process | extracellular structure organization | 0 | 0 | 0.000724 |
| GO:0043066 | biological_process | negative regulation of apoptotic process | 0.000038 | 0.000009 | 0.000257 |
| GO:0043067 | biological_process | regulation of programmed cell death | 0 | 0.000001 | 0 |
| GO:0043069 | biological_process | negative regulation of programmed cell death | 0.000023 | 0.000006 | 0.000141 |
| GO:0043085 | biological_process | positive regulation of catalytic activity | 0 | 0.000085 | 0.000027 |
| GO:0043235 | cellular_component | receptor complex | 0 | 0.000009 | 0.000662 |
| GO:0043408 | biological_process | regulation of MAPK cascade | 0 | 0.000049 | 0.000168 |
| GO:0043410 | biological_process | positive regulation of MAPK cascade | 0 | 0.000205 | 0.0007 |
| GO:0044092 | biological_process | negative regulation of molecular function | 0 | 0.000076 | 0.000386 |
| GO:0044117 | biological_process | growth of symbiont in host | 0.000355 | 0.000108 | 0.000374 |
| GO:0044130 | biological_process | negative regulation of growth of symbiont in host | 0.000114 | 0.000447 | 0.00019 |
| GO:0044146 | biological_process | negative regulation of growth of symbiont involved in interaction with host | 0.000114 | 0.000447 | 0.00019 |
| GO:0044421 | cellular_component | extracellular region part | 0 | 0 | 0 |
| GO:0044459 | cellular_component | plasma membrane part | 0 | 0 | 0 |
| GO:0045087 | biological_process | innate immune response | 0 | 0 | 0 |
| GO:0045088 | biological_process | regulation of innate immune response | 0 | 0.000425 | 0.000285 |
| GO:0045121 | cellular_component | membrane raft | 0.000046 | 0.000011 | 0.000111 |
| GO:0045321 | biological_process | leukocyte activation | 0 | 0 | 0.000005 |
| GO_ID | Category | Name | *P*  (B-ALL vs AML) | *P*  (B-ALL vs MPAL) | *P*  (AML vs  MPAL) |
| GO:0046649 | biological_process | lymphocyte activation | 0 | 0 | 0.000246 |
| GO:0046903 | biological_process | secretion | 0 | 0.000004 | 0.000091 |
| GO:0048513 | biological_process | organ development | 0 | 0.000001 | 0 |
| GO:0048514 | biological_process | blood vessel morphogenesis | 0 | 0.000006 | 0.000027 |
| GO:0048523 | biological_process | negative regulation of cellular process | 0 | 0 | 0.000013 |
| GO:0048856 | biological_process | anatomical structure development | 0 | 0 | 0.000003 |
| GO:0048870 | biological_process | cell motility | 0 | 0 | 0 |
| GO:0050663 | biological_process | cytokine secretion | 0 | 0.000001 | 0.000001 |
| GO:0050707 | biological_process | regulation of cytokine secretion | 0 | 0.000007 | 0.00005 |
| GO:0050708 | biological_process | regulation of protein secretion | 0 | 0.000009 | 0.000302 |
| GO:0050715 | biological_process | positive regulation of cytokine secretion | 0.000009 | 0.000178 | 0.000103 |
| GO:0050727 | biological_process | regulation of inflammatory response | 0 | 0 | 0.00072 |
| GO:0050776 | biological_process | regulation of immune response | 0 | 0 | 0 |
| GO:0050778 | biological_process | positive regulation of immune response | 0 | 0 | 0.000085 |
| GO:0050790 | biological_process | regulation of catalytic activity | 0 | 0.000018 | 0 |
| GO:0050793 | biological_process | regulation of developmental process | 0 | 0.000073 | 0 |
| GO:0050830 | biological_process | defense response to Gram-positive bacterium | 0 | 0 | 0.000609 |
| GO:0050865 | biological_process | regulation of cell activation | 0 | 0 | 0.000012 |
| GO:0050867 | biological_process | positive regulation of cell activation | 0 | 0.000021 | 0.000454 |
| GO:0050896 | biological_process | response to stimulus | 0 | 0 | 0 |
| GO:0050900 | biological_process | leukocyte migration | 0 | 0 | 0.000018 |
| GO:0051050 | biological_process | positive regulation of transport | 0 | 0.000066 | 0.000075 |
| GO_ID | Category | Name | *P*  (B-ALL vs AML) | *P*  (B-ALL vs MPAL) | *P*  (AML vs  MPAL) |
| GO:0051128 | biological_process | regulation of cellular component organization | 0 | 0.00002 | 0.00014 |
| GO:0051246 | biological_process | regulation of protein metabolic process | 0 | 0.000179 | 0.000019 |
| GO:0051247 | biological_process | positive regulation of protein metabolic process | 0 | 0.000723 | 0.000019 |
| GO:0051270 | biological_process | regulation of cellular component movement | 0 | 0.000008 | 0 |
| GO:0051272 | biological_process | positive regulation of cellular component movement | 0 | 0.000252 | 0.000001 |
| GO:0051347 | biological_process | positive regulation of transferase activity | 0 | 0.000713 | 0.000014 |
| GO:0051604 | biological_process | protein maturation | 0.000898 | 0.000041 | 0.000796 |
| GO:0051707 | biological_process | response to other organism | 0 | 0 | 0 |
| GO:0051716 | biological_process | cellular response to stimulus | 0 | 0 | 0 |
| GO:0052547 | biological_process | regulation of peptidase activity | 0 | 0.000013 | 0.000187 |
| GO:0055082 | biological_process | cellular chemical homeostasis | 0.000002 | 0.000167 | 0.000496 |
| GO:0060326 | biological_process | cell chemotaxis | 0 | 0 | 0.000006 |
| GO:0060548 | biological_process | negative regulation of cell death | 0.000004 | 0.000011 | 0.000051 |
| GO:0070887 | biological_process | cellular response to chemical stimulus | 0 | 0 | 0 |
| GO:0071219 | biological_process | cellular response to molecule of bacterial origin | 0 | 0.000001 | 0.000003 |
| GO:0071222 | biological_process | cellular response to lipopolysaccharide | 0 | 0.000002 | 0.000002 |
| GO:0071310 | biological_process | cellular response to organic substance | 0 | 0.000001 | 0 |
| GO:0071363 | biological_process | cellular response to growth factor stimulus | 0.000013 | 0.000967 | 0.000862 |
| GO:0071396 | biological_process | cellular response to lipid | 0.000005 | 0.000229 | 0.000037 |
| GO:0071944 | cellular_component | cell periphery | 0 | 0 | 0 |
| GO:0072358 | biological_process | cardiovascular system development | 0 | 0.000023 | 0.000006 |
| GO_ID | Category | Name | *P*  (B-ALL vs AML) | *P*  (B-ALL vs MPAL) | *P*  (AML vs  MPAL) |
| GO:0072359 | biological_process | circulatory system development | 0 | 0.000023 | 0.000006 |
| GO:0080134 | biological_process | regulation of response to stress | 0 | 0 | 0 |
| GO:2000145 | biological_process | regulation of cell motility | 0 | 0.000016 | 0 |
| GO:2000147 | biological_process | positive regulation of cell motility | 0 | 0.00016 | 0 |

**Table S3**.

Gene sets with FDR less than 0.25 and a nominal p-value less than 0.01 in GSEA of AML and B-ALL.

| Category | Signaling pathway | NES | NOM p-val | FDR q-val |
| --- | --- | --- | --- | --- |
| AML | KEGG_LYSOSOME | 2.57 | <0.001 | <0.001 |
|  | KEGG_TOLL_LIKE_RECEPTOR_SIGNALING_PATHWAY | 2.27 | <0.001 | <0.001 |
|  | KEGG_CYTOKINE_CYTOKINE_RECEPTOR_INTERACTION | 2.25 | <0.001 | <0.001 |
|  | KEGG_OTHER_GLYCAN_DEGRADATION | 2.20 | <0.001 | <0.001 |
|  | KEGG_GLYCOSAMINOGLYCAN_DEGRADATION | 2.19 | <0.001 | <0.001 |
|  | KEGG_CYTOSOLIC_DNA_SENSING_PATHWAY | 2.18 | <0.001 | <0.001 |
|  | KEGG_NATURAL_KILLER_CELL_MEDIATED_CYTOTOXICITY | 2.15 | <0.001 | <0.001 |
|  | KEGG_HEMATOPOIETIC_CELL_LINEAGE | 2.15 | <0.001 | <0.001 |
|  | KEGG_NOD_LIKE_RECEPTOR_SIGNALING_PATHWAY | 2.12 | <0.001 | <0.001 |
|  | KEGG_STARCH_AND_SUCROSE_METABOLISM | 2.00 | <0.001 | 0.002 |
|  | KEGG_DRUG_METABOLISM_OTHER_ENZYMES | 1.99 | <0.001 | 0.002 |
|  | KEGG_LEISHMANIA_INFECTION | 1.92 | <0.001 | 0.004 |
|  | KEGG_APOPTOSIS | 1.90 | 0.002 | 0.005 |
|  | KEGG_CHEMOKINE_SIGNALING_PATHWAY | 1.85 | <0.001 | 0.009 |
|  | KEGG_AMINO_SUGAR_AND_NUCLEOTIDE_SUGAR_METABOLISM | 1.83 | <0.001 | 0.010 |
|  | KEGG_HISTIDINE_METABOLISM | 1.78 | <0.001 | 0.015 |
|  | KEGG_O_GLYCAN_BIOSYNTHESIS | 1.76 | 0.004 | 0.019 |
|  | KEGG_FRUCTOSE_AND_MANNOSE_METABOLISM | 1.72 | 0.003 | 0.028 |
|  | KEGG_FC_GAMMA_R_MEDIATED_PHAGOCYTOSIS | 1.70 | 0.002 | 0.029 |
|  | KEGG_GLYCOLYSIS_GLUCONEOGENESIS | 1.70 | 0.005 | 0.028 |
|  | KEGG_NEUROACTIVE_LIGAND_RECEPTOR_INTERACTION | 1.69 | <0.001 | 0.029 |
|  | KEGG_EPITHELIAL_CELL_SIGNALING_IN_HELICOBACTER_PYLORI_INFECTION | 1.69 | 0.002 | 0.029 |
| Category | Signaling pathway | NES | NOM p-val | FDR q-val |
| AML | KEGG_LEUKOCYTE_TRANSENDOTHELIAL_MIGRATION | 1.64 | <0.001 | 0.042 |
|  | KEGG_FC_EPSILON_RI_SIGNALING_PATHWAY | 1.63 | 0.003 | 0.045 |
|  | KEGG_RIG_I_LIKE_RECEPTOR_SIGNALING_PATHWAY | 1.63 | 0.002 | 0.043 |
|  | KEGG_PORPHYRIN_AND_CHLOROPHYLL_METABOLISM | 1.63 | 0.006 | 0.042 |
|  | KEGG_JAK_STAT_SIGNALING_PATHWAY | 1.61 | 0.002 | 0.045 |
|  | KEGG_T_CELL_RECEPTOR_SIGNALING_PATHWAY | 1.58 | 0.006 | 0.055 |
|  | KEGG_MAPK_SIGNALING_PATHWAY | 1.41 | 0.005 | 0.162 |
| B-ALL | KEGG_RIBOSOME | 2.66 | <0.001 | <0.001 |
|  | KEGG_SPLICEOSOME | 1.88 | <0.001 | 0.020 |

NES, normalized enrichment score; NOM p-val, nominal p-value; FDR q-val, false discovery rate q-value

**Table S4.**

Predictive performance of the scoring model and that of the SVM model in 12 samples and public gene expression data.

(A) 12 samples applied to the scoring model

|  | AML (n=3) | B-ALL (n=5) | MPAL (n=4) | PPV | NPV |
| --- | --- | --- | --- | --- | --- |
| predicted AML | 3 | 0 | 1 | 75.0% | 100.0% |
| predicted B-ALL | 0 | 5 | 0 | 100.0% | 85.7% |
| predicted MPAL | 0 | 0 | 3 | 100.0% | 88.9% |
| Sensitivity | 100.0% | 100.0% | 75.0% |  |  |
| Specificity | 88.9% | 85.7% | 100.0% |  |  |

(B) public gene expression data applied to the scoring model

|  | AML (n=197) | B-ALL (n=206) | MPAL (n=24) | PPV | NPV |
| --- | --- | --- | --- | --- | --- |
| predicted AML | 188 | 2 | 1 | 98.4% | 96.2% |
| predicted B-ALL | 7 | 204 | 0 | 96.7% | 99.1% |
| predicted MPAL | 2 | 0 | 23 | 92.0% | 99.8% |
| Sensitivity | 95.4% | 99.0% | 95.8% |  |  |
| Specificity | 98.7% | 95.5% | 97.3% |  |  |

(C) 12 samples applied to the SVM model

|  | AML (n=3) | B-ALL (n=5) | MPAL (n=4) | PPV | NPV |
| --- | --- | --- | --- | --- | --- |
| predicted AML | 3 | 0 | 0 | 100.0% | 100.0% |
| predicted B-ALL | 0 | 5 | 0 | 100.0% | 100.0% |
| predicted MPAL | 0 | 0 | 4 | 100.0% | 100.0% |
| Sensitivity | 100.0% | 100.0% | 100.0% |  |  |
| Specificity | 100.0% | 100.0% | 100.0% |  |  |

(D) public gene expression data applied to the SVM model

|  | AML (n=197) | B-ALL (n=206) | MPAL (n=24) | PPV | NPV |
| --- | --- | --- | --- | --- | --- |
| predicted AML | 196 | 1 | 2 | 98.5% | 99.6% |
| predicted B-ALL | 0 | 205 | 0 | 100.0% | 99.5% |
| predicted MPAL | 1 | 0 | 22 | 95.7% | 99.5% |
| Sensitivity | 99.5% | 99.5% | 91.7% |  |  |
| Specificity | 98.7% | 100.0% | 99.8% |  |  |
